# Survival and cost-effectiveness of helicopter versus ground emergency medical services: a systematic review and meta-analysis with meta-regression and trial sequential analysis

**DOI:** 10.1186/s13049-025-01478-0

**Published:** 2025-10-03

**Authors:** Daniele Orso, Luca Flaibani, Ugo Giulio Sisto, Marco Bonsano, Federico Fonda, Rocco Pangallo, Tiziana Bove

**Affiliations:** 1 Department of Emergency, University Hospital of Udine, Azienda Sanitaria Universitaria Friuli Centrale, “Santa Maria della Misericordia”, Piazzale Santa Maria della Misericordia 15, Udine, 33100 UD Italy; 2 Department of Emergency, Ospedale di Cattinara, University Hospital of Trieste, Azienda Sanitaria Giuliano Isontina, Strada di Fiume 447, Trieste, 34149 Italy; 3 Department of Medicine, University of Udine, Via Colugna 51, Udine, 33100 Italy

**Keywords:** HEMS, GEMS, Helicopter, Emergency medical system, Mortality, Meta-analysis

## Abstract

**Objective:**

To synthesise the available literature comparing outcomes of ground emergency medical services (GEMS) and helicopter emergency medical services (HEMS).

**Methods:**

We conducted a systematic review and meta-analysis, reported in accordance with the Preferred Reporting Items for Systematic Reviews and Meta-Analyses (PRISMA) guidelines. PubMed, Scopus, Web of Science, and the Cumulative Index to Nursing and Allied Health Literature (CINAHL) were searched from 1995 to 2024. Studies comparing HEMS with GEMS in emergency conditions were eligible.

**Results:**

The search retrieved 1,595 records; 181 studies were assessed in full text, and 77 were included, accounting for a pooled population of 2,618,483 patients. The relative risk (RR) of mortality in HEMS compared with GEMS was 1.13 (95% CI 0.96–1.34). The RR of disability was 1.24 (95% CI 0.99–1.55). The total incremental net benefit was €980,000 per QALY per patient, based on cost-effectiveness studies and a willingness-to-pay threshold of €35 million per QALY per patient.

**Conclusion:**

Very low-quality evidence, due to high heterogeneity, potential confounding from registry-based enrolment, and possible multiple imputation bias, suggested that HEMS did not improve survival compared with GEMS. High-quality studies are needed to further investigate this question.

**Clinical trial registration:**

PROSPERO: International prospective register of systematic reviews, 2024, CRD42024628317.

**Supplementary Information:**

The online version contains supplementary material available at 10.1186/s13049-025-01478-0.

## Introduction

Emergency Medical Services (EMS) are instrumental in saving lives by providing rapid medical assistance and transport in out-of-hospital settings. Traditionally, Ground Emergency Medical Services (GEMS) have been the standard response, using ambulances to deliver care. However, in situations requiring time-sensitive interventions, and in rural or remote areas, Helicopter Emergency Medical Services (HEMS) have emerged as an alternative, offering faster access to healthcare resources, particularly in cases of trauma, cardiac events, or severe medical emergencies [1, 2].

HEMS involves the use of helicopters to transport patients from the site of injury or illness to appropriate medical facilities and can potentially reduce transport time substantially. It is commonly believed that the speed and accessibility of HEMS increase survival rates, particularly in time-sensitive conditions, whereas GEMS is regarded as a more cost-effective and widely available solution, but may be limited by traffic, distance, and terrain [3].

Although several studies have examined the effectiveness of HEMS versus GEMS, the evidence remains inconclusive regarding which service provides better outcomes in different settings, such as trauma, stroke, or acute coronary syndrome. This systematic review aimed to evaluate and synthesise the available literature to compare outcomes between HEMS and GEMS, with a focus on patient survival and healthcare costs. The objective was to provide a comprehensive assessment of the strengths and weaknesses of both systems and to offer insights for policymakers, healthcare providers, and emergency response teams.

## Material and method

We conducted a systematic review and meta-analysis of the literature. The protocol was prospectively registered in the International Prospective Register of Systematic Reviews (PROSPERO; CRD42024628317, 2024), and the review was reported in accordance with the Preferred Reporting Items for Systematic Reviews and Meta-Analyses (PRISMA) guidelines [4]. Ethical approval by the local Institutional Review Board was not required because the study was based on data from previously approved studies.

### Eligibility criteria, search strategy and data collection

We included studies that compared HEMS and GEMS in emergency health conditions. Eligible designs were randomised controlled trials, prospective or retrospective observational studies, retrospective studies with propensity score matching, and interventional studies. No language restrictions were applied.

We excluded editorials, comments, letters to the editor, conference abstracts, case reports, clinical guidelines, and literature reviews with or without meta-analysis. Studies involving animals and those not reporting outcome data were also excluded. Because of substantial advances in prehospital care, technologies, and protocols over the past 30 years, we excluded studies published before 1994, applying a 30-year time frame.

We searched PubMed, Scopus, Web of Science, and the Cumulative Index to Nursing and Allied Health Literature (CINAHL) from Jan 1, 1995, to Oct 25, 2024. To ensure a comprehensive synthesis of the available literature, we also retrieved and analysed existing meta-analyses on the same topic during the screening phase to identify relevant studies for inclusion.

One researcher (DO) created search strings for each database. These search strings were peer reviewed before execution [5] by an experienced researcher (FF), following the Peer Review of Electronic Search Strategies (PRESS) checklist [6]. Search results were imported into the Covidence platform (Veritas Health Innovation Ltd, 446 Collins St, Melbourne, VIC 3000, Australia). The keywords used were ‘HEMS’ and ‘GEMS’ (in their various extensions and forms). Full details of the search strings are provided in the Supplementary Material (Table 1S).

The selection process involved two phases: title and abstract screening, followed by full-text screening. After removal of duplicates, three researchers (DO, TB, and UGS) independently and blindly screened the studies. Conflicts were resolved through discussion until consensus was reached on article eligibility.

The following data were extracted: author name(s), year, dispatch, study design, time frame of case records, country, case registry used, sample size, type of mortality considered, number of patients with an Injury Severity Score (ISS) ≥ 15 in each group, median ISS value for each group, number of deaths in the intervention and control groups, and disability rate in each group. An electronic data extraction form was developed in the Covidence platform and piloted with at least three selected articles to ensure its usefulness, appropriateness, and feasibility [5, 7]. Data were extracted independently by two reviewers (DO and FF), both of whom were trained and had appropriate knowledge of the topic. Conflicts were resolved through discussion until consensus was reached, and the final decision was made after consensus was established.

### Risk of bias assessment

Two authors (DO and FF) independently assessed the risk of bias. We used ROBINS-E (Risk of Bias in Non-Randomised Studies of Exposure) [8], considering HEMS and GEMS not as interventions per se but as system-level exposures, reflecting logistical and organisational models to which patients were allocated by regional protocols rather than randomisation. Discrepancies were resolved through discussion until consensus was reached.

### Assessing the quality of evidence

Two reviewers (DO and FF) independently applied the Grading of Recommendations Assessment, Development, and Evaluation (GRADE) approach [9] in duplicate to assess the quality of evidence for each outcome. We used the GRADEpro software (GRADEpro GDT; GRADEpro Guideline Development Tool [Software], McMaster University, 1280 Main Street West, Hamilton, Ontario) to generate the evidence profile.

### Statistical analysis

For the meta-analysis, binary outcomes (number of events in each group) were identified. We conducted both fixed-effect and random-effects analyses. In the fixed-effect model, relative risks (RRs) were calculated using the Mantel–Haenszel method; in the random-effects model, the inverse-variance method was applied. A Bayesian hierarchical model was used as a sensitivity analysis for the main results. As an additional sensitivity analysis, we excluded studies that derived data from the same registry with overlapping time frames, retaining only the study with the largest sample size, and re-ran the random-effects model to assess robustness. Between-study inconsistency was assessed with the I² statistic, and forest plots were used to present the meta-analysis findings.

We focused on outliers to identify potential causes of heterogeneity. We conducted an influence analysis to determine the most influential studies contributing to heterogeneity. Subgroup analyses were performed according to study characteristics (i.e., prospective, propensity score–matched, and retrospective). To further explore sources of heterogeneity, we conducted a meta-regression (mixed-effects model) using prespecified study-level features: dispatch type, HEMS crew composition (physician vs. paramedic), number of patients with ISS ≥ 15 in each group, median ISS values, and the time frame of the case registry. A meta-CART (classification and regression tree) model was also performed to analyse moderators associated with heterogeneity.

Publication bias was evaluated with a funnel plot and the Egger test. A trial sequential analysis was conducted to estimate statistical power and required sample size.

Comparative efficiency research (COMER) was conducted to estimate the total incremental net benefit (TINB), weighting the incremental net benefit (INB) of each included study by the inverse of its variance, according to the method described by Crespo et al. [10].

All analyses were performed in R (version 4.3.3; R Foundation for Statistical Computing, Vienna, Austria) using the following packages: *meta*,* dmetar*,* tidyverse*,* metafor*,* ggplot2*,* gridExtra*,* robvis*,* esc*,* brms*,* forestplot*,* irr*,* RTSA*,* forecast*,* stringr*,* dplyr*,* ggridges*,* glue*,* and metacart*.

## Results

### Study selection

**Fig. 1:**
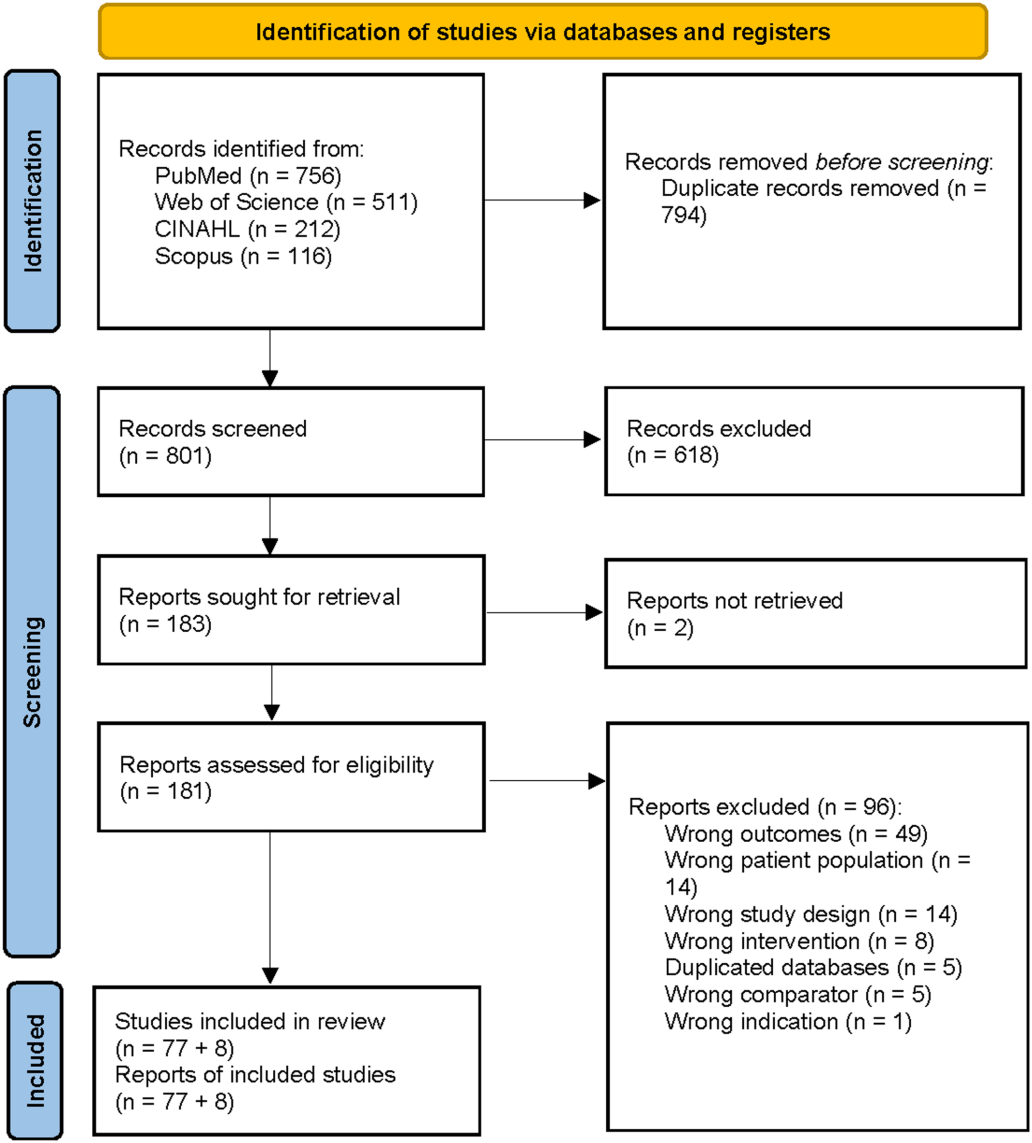
PRISMA flowchart. One hundred and eighty-one records were found during the initial identification process. Seventy-seven studies were finally included (and eight studies about cost-effectiveness analysis) and analyzed in the systematic review and quantitative synthesis, accounting for a pooled population of 2,097,650 patients

The initial search identified 1,595 records. After removal of non-relevant and duplicate records, 181 studies were assessed in full-text analysis (Fig. 1). The systematic review and quantitative synthesis included 77 studies, accounting for a pooled population of 2,618,483 patients [11–77]. Eight additional studies were included only in the cost-effectiveness analysis [78–85]. Agreement between reviewers is reported in the Supplementary Material (Table 2 S).

### Characteristics of the studies included

Table 1 shows the main characteristics of the included studies, published between 2002 and 2024. All but 22 studies were retrospective; of these 22, 15 used propensity-score matching and seven were prospective.

**Table 1:** Characteristics of the studies included in the systematic review. PSM: Propensity Score Matching; NS: not specified; ISS: Injury Severity Score; HEMS: helicopter emergency medical system; GEMS: ground emergency medical system; Survival in groups: percentage of patients survived; Disability in groups: percentage of patients with disability at the discharge; RoB: overall risk of bias

Id	Study	Dispatch	Type	Time frame	Country	Registry	Sample Size	HEMS crew leader	GEMS crew leader	Mortality	ISS ≥ 15 in HEMS group (%)	ISS ≥ 15 in GEMS group (%)	median ISS in HEMS group	median ISS in GEMS group	Survival in HEMS group (%)	Survival in GEMS group (%)	Disability in HEMS group (%)	Disability in GEMS group (%)	RoB
1	Abe et al. 2014 [11]	Trauma	Prospective	2004–2011	Japan	Japan Trauma Data Bank	24,293	physician	paramedic	Overall	90	91			74	74	51	41	High
2	Ageron et al. 2020 [12]	Trauma	Retrospective	2009–2017	France	Trauma system of the Northern French Alps Emergency Network (TRENAU) Registry	9,458	physician	paramedic	Overall	47	43	16	16	94	93			High
3	Aiolfi et al. 2018 [13]	Traumatic brain injury	Retrospective	2007–2014	USA	National Trauma Data Bank (NTDB) by the American College of Surgeons Committee on Trauma	145,559			30-day					88	92			High
4	Alsrtup et al. 2021 [14]	Mixed	Retrospective	2014–2018	Denmark	Danish Civil Registration System	2,575	physician	paramedic/physician	30-day					78	77			Some concerns
5	Al-Thani et al. 2017 [15]	Trauma	Retrospective	2011–2013	Qatar	Hamad Trauma Center Registry	4,596	paramedic	paramedic	In-hospital	50	26	15	10	90	96			High
6	Andruszkow et al. 2016 [16]	Trauma	Retrospective	2002–2012	Germany	Trauma Register DGU^®^ (TR-DGU) of the German Trauma Society	52,281	physician	physician	Overall	75	65	25	22	86	87			High
7	Beaumont et al. 2020 [17]	Trauma	Propensity	2012–2017	England	UK Trauma Audit and Research Network registry	9,272	paramedic/physician	paramedic	Overall			20	10	87	90			Some concerns
8	Bekelis et al. 2015 [18]	Traumatic brain injury	Retrospective	2009–2011	Lebanon	National Trauma Data Bank (NTDB) by the American College of Surgeons Committee on Trauma	127,831			Overall	31	16			88	92			High
9	Berlot et al. 2009 [19]	Traumatic brain injury	Retrospective	2002–2007	Italy	Trieste General Hospital registry	194	physician	paramedic/physician	Overall			29	26	79	75			High
10	Bläsius et al. 2021 [20]	Pediatric trauma	Retrospective	2015–2020	Germany	Trauma Register DGU^®^ (TR-DGU) of the German Trauma Society	2,755	physician	physician	Overall			21	18	92	92			High
11	Brown et al. 2016 [21]	Trauma	Propensity	2007–2012	USA	National Trauma Data Bank (NTDB) by the American College of Surgeons Committee on Trauma	311,382			In-hospital			13	9	92	93			Some concerns
12	Brown et al. 2016 2nd [22]	Pediatric trauma	Propensity	2007–2012	USA	National Trauma Data Bank (NTDB) by the American College of Surgeons Committee on Trauma	51,400			In-hospital	30	30	9	9	96	94	9	8	Some concerns
13	Buchanan et al. 2016 [23]	Trauma	Retrospective	1995–2013	Canada	Hamilton General Hospital Trauma Registry	3,146	physician	paramedic	Overall	67	52			91	84			Some concerns
14	Bulger et al. 2012 [24]	Trauma	Prospective	2006–2009	USA/Canada	Resuscitation Outcomes Consortium RCTs	2,049			In-hospital			30	23	73	77			Some concerns
15	Chen et al. 2013 [25]	Trauma	Propensity	2000–2013	USA	Pennsylvania state trauma registry	16,614			In-hospital			14	11	90	89			Some concerns
16	Ciaraglia et al. 2024 [26]	Pediatric trauma	Propensity	2007–2016	USA	National Trauma Data Bank (NTDB) by the American College of Surgeons Committee on Trauma	59,502			In-hospital			10	10	97	97	16	14	Some concerns
17	Colnaric et al. 2021 [27]	Trauma	Propensity	2015–2017	USA	National Trauma Data Bank (NTDB) by the American College of Surgeons Committee on Trauma	1,898			In-hospital	20	20			91	88			Some concerns
18	Davis et al. 2015 [28]	Traumatic brain injury	Retrospective	1987–2003	USA	San Diego County Trauma Registry	10,314	physician	paramedic	Overall			28	26	75	75	42	42	High
19	Di Bartolomeo et al. 2001 [29]	Traumatic brain injury	Prospective	1998–1999	Italy	Friuli Venezia Giulia Major Trauma Outcome Study	184	physician	paramedic	30-day	100	100	33	30	70	76			Some concerns
20	Dominguez et al. 2020 [30]	Trauma	Retrospective	2010–2016	USA	American College of Surgeons Trauma Quality Improvement Program	723,483			Overall			17	10	91	95			High
21	Duffens et al. 2022 [31]	Pediatric trauma	Retrospective	2014–2016	USA	Pediatric Trauma Quality Improvement Program	2,090			Overall			10	5	87	90			High
22	Elkbuli et al. 2022 [32]	Trauma	Retrospective	2013–2018	USA	South Florida First Level Trauma Center Registry	12,633	paramedic	paramedic	Overall					93	97			High
23	Englum et al. 2017 [33]	Pediatric trauma	Retrospective	2007–2011	USA	National Trauma Data Bank (NTDB) by the American College of Surgeons Committee on Trauma	37,373			Overall	55	36			93	95			High
24	Enomoto et al. 2020 [34]	Pediatric trauma	Retrospective	2004–2015	Japan	Japan Trauma Data Bank	5,947	physician	paramedic	In-hospital	7	4	16	9	96	99	33	15	High
25	Farach et al. 2018 [35]	Pediatric trauma	Retrospective	2000–2012	USA	All Children’s Hospital Johns Hopkins Medicine trauma registry	1,709	paramedic	paramedic	In-hospital					97	99	8	2	High
26	Funder et al. 2017 [36]	Stroke	Prospective	2010–2013	Denmark	Danish Stroke Registry	1,068	physician	paramedic/physician	30-day					92	93	42	41	High
27	Funder et al. 2018 [37]	Acute Coronary Syndrome	Prospective	2010–2013	Denmark	Rigshospitalet PCI Registry	1,604	physician	paramedic/physician	30-day					95	94	23	26	Some concerns
28	Galvagno et al. 2012 [38]	Trauma	Retrospective	2007–2009	USA	National Trauma Data Bank (NTDB) by the American College of Surgeons Committee on Trauma	159,511			Overall	100	100			87	89	38	30	Some concerns
29	Günkel et al. 2015 [39]	Trauma	Retrospective	2009–2009	Switzerland	Universitäts Spital Zürich Registry	365	physician	paramedic/physician	Overall			26	20	81	82			High
30	Hakakian et al. 2019 [40]	Trauma	Retrospective	2016–2016	USA	National Trauma Data Bank (NTDB) by the American College of Surgeons Committee on Trauma	903			Overall			10	7	93	94			High
31	Hannay et al. 2014 [41]	Trauma	Retrospective	1998–2008	USA	Emory University Trauma Service Registry	13,802			In-hospital	55	16	17	10	85	88			High
32	Hata et al. 2006 [42]	Acute Coronary Syndrome	Retrospective	2002–2003	Japan	Chiba Hokusoh Hospital Internal Registry	76	physician	physician	In-hospital					95	89			Very high
33	Herrlin Jensen et al. 2021 [43]	Mixed	Retrospective	2014–2016	Denmark	five Danish regions EMS dispatch centers registry	593	physician	paramedic/physician	30-day					93	94			Some concerns
34	Hesselfeldt et al. 2013 [44]	Trauma	Prospective	2009–2011	Denmark	Internal Registry	204	physician	paramedic/physician	30-day			26	25	77	71			High
35	Hesselfeldt et al. 2014 [45]	Stroke	Prospective	2010–2011	Denmark	Internal Registry	109	physician	paramedic/physician	30-day			10	8	100	91			Some concerns
36	Hosomi et al. 2022 [46]	Traumatic brain injury	Propensity	2004–2018	Japan	Japan Trauma Data Bank	7,420	physician	paramedic	In-hospital			26	26	81	79	6	7	High
37	Ishikura et al. 2021 [47]	Acute Coronary Syndrome	Retrospective	2015–2018	Japan	Japan Helicopter Emergency Medical Service Registry	871	physician	paramedic	Overall					95	93			Some concerns
38	Ishiyama et al. 2021 [48]	Acute Coronary Syndrome	Retrospective	2013–2017	Japan	Mie Acute Coronary Syndrome Registry	106	physician	paramedic	In-hospital					98	93			Some concerns
39	Jitsuiki et al. 2022 [49]	Severe Abdominal Trauma	Propensity	2004–2019	Japan	Japan Trauma Data Bank	2,785	physician	paramedic	Overall			27	28	78	80			High
40	Kashyap et al. 2016 [50]	Sepsis	Retrospective	2007–2009	USA	Internal Registry	181			In-hospital			9	7	70	83			High
41	Kim et al. 2015 [51]	Trauma	Retrospective	2011–2014	Korea	Internal Registry	1,626	physician	paramedic	Overall			9	9	95	91			High
42	Knobloch et al. 2009 [52]	Aortic dissection	Retrospective	1996–2005	Germany	Internal Registry	130	physician	physician	Overall					8	78			Very high
43	Kushida et al. 2021 [53]	Severe Thoracic Trauma	Propensity	2004–2019	Japan	Japan Trauma Data Bank	14,656	physician	paramedic	Overall			28	27	84	81			Some concerns
44	Lee et al. 2020 [54]	Stroke	Retrospective	2013–2015	Korea	National Emergency Database Information System	128	physician	paramedic	Overall					98	93			High
45	Lee et al. 2023 [55]	Trauma	Retrospective	2013–2014	Korea	National Emergency Database Information System	139	physician	paramedic	Overall	100	100	22	23	91	78			High
46	McCowan et al. 2006 [56]	Trauma	Retrospective	1997–2001	USA	Regional Trauma Registry	575	paramedic	paramedic	In-hospital	23	14	11	9	98	100	8	3	High
47	Michaels et al. 2019 [57]	Trauma	Retrospective	2014–2014	USA	National Trauma Data Bank (NTDB) by the American College of Surgeons Committee on Trauma	469,407			Overall			16	11	94	97			High
48	Missios et al. 2014 [58]	Pediatric TBI	Retrospective	2009–2011	USA	National Trauma Data Bank (NTDB) by the American College of Surgeons Committee on Trauma	11,309			In-hospital	21	9			93	96			High
49	Mitchell et al. 2007 [59]	Trauma	Retrospective	1998–2002	Canada	Nova Scotia Provincial Trauma Registry	823	paramedic	paramedic	Overall			25	20	82	82			High
50	Nabeta et al. 2021 [60]	Trauma	Propensity	2014–2018	Japan	Internal Registry	208	physician	paramedic	24-hour	100	100			86	86			High
51	Nishigoori et al. 2022 [61]	Acute Coronary Syndrome	Propensity	2008–2015	Japan	Nippon Medical School Chiba Hokusoh Hospital Registry	324			In-hospital					94	85			High
52	Ota et al. 2021 [62]	Traumatic brain injury	Retrospective	2004–2019	Japan	Japan Trauma Data Bank	41,358	physician	paramedic	Overall			16	16	80	86			High
53	Polites et al. 2017 [63]	Pediatric trauma	Propensity	2010–2011	USA	National Trauma Data Bank (NTDB) by the American College of Surgeons Committee on Trauma	4,484			In-hospital					91	89			Some concerns
54	von Recklinghausen 2011 [64]	Trauma	Retrospective	2003–2008	USA	Internal Registry	2,164			Overall			16	9	94	95			High
55	Ryb et al. 2013 [65]	Trauma	Retrospective	2007–2007	USA	National Trauma Data Bank (NTDB) by the American College of Surgeons Committee on Trauma	192,422			Overall					94	96			High
56	Schneider et al. 2021 [66]	Trauma	Retrospective	2014–2017	USA	Internal Registry	3,967			In-hospital			17	9	87	93			High
57	Stassen et al. 2020 [67]	Trauma	Propensity	2017–2018	South Africa	Internal Registry	410	physician	paramedic	30-day			17	9	97	98			Some concerns
58	Stewart et al. 2015 [68]	Pediatric trauma	Retrospective	2003–2013	USA	Internal Registry	14,405			Overall	44	12			89	99			Some concerns
59	Stowell et al. 2019 [69]	Mixed	Retrospective	2014–2014	France	SAMU registry	239	physician	physician	28-day					88	97	8	3	High
60	Sun et al. 2017 [70]	Traumatic brain injury	Retrospective	2007–2014	USA	National Trauma Data Bank (NTDB) by the American College of Surgeons Committee on Trauma	1,018			In-hospital	6	6	8	8	98	99			High
61	Talving et al. 2009 [71]	Trauma	Retrospective	1998–2007	USA	Trauma registry of the LosAngeles County + University of Southern California (LAC + USC) MedicalCenter	3,373			Overall	26	19	11	9	98	99			High
62	Taniguchi et al. 2024 [72]	Aortic dissection	Retrospective	2015–2020	Japan	Japanese Society for Aeromedical Services registry	342			In-hospital					81	76	23	33	High
63	Thomas et al. 2002 [73]	Trauma	Retrospective	1995–1998	USA	Internal Registry	16,699	paramedic	paramedic	Overall	42	12			91	97			High
64	Tsuchiya et al. 2016 [74]	Trauma	Propensity	2004–2014	Japan	Japan Trauma Data Bank	7,960	physician	paramedic	In-hospital	56	54			78	76	23	24	Some concerns
65	Ueno et al. 2019 [75]	Stroke	Retrospective	2014–2017	Japan	Internal Registry	1,216	physician	paramedic	Overall			13	7	81	90			High
66	Zadorozny et al. 2024 [76]	Trauma	Retrospective	2017–2022	USA	Trauma Quality Improvement Program	34,504			In-hospital					90	90			High
67	Zhu et al. 2018 [77]	Trauma	Propensity	1999–2012	USA	Internal Registry	1,049	paramedic	paramedic	Overall	50	26			96	92			Some concerns

Five studies included more than 150,000 patients [21, 30, 38, 57, 65]; the largest, by Dominguez et al., included about 720,000 patients [30], whereas the smallest, by Hata et al., included 76 patients [42].

The USA and Japan were the most represented countries. In the USA, helicopter rescues were mostly staffed by paramedics, whereas in Japan, they were almost exclusively staffed by physicians.

Of the 77 studies selected, 34 included trauma patients [11, 12, 15–17, 21, 23–25, 27, 30, 32, 38–41, 44, 49, 51, 53, 55–57, 59, 60, 64–67, 71, 73, 74, 76, 77]; eight focused on traumatic brain injury (TBI) [13, 18, 19, 28, 29, 46, 62, 70]; ten on paediatric trauma [20, 22, 26, 31, 33–35, 58, 63, 68]; and 12 on medical emergencies, including five on acute coronary syndrome (ACS) [37, 42, 47, 48, 61], two on suspected acute aortic dissection [52, 72], four on stroke [36, 45, 54, 75], and one on sepsis [50]. Three studies included mixed populations [14, 43, 69].

Only a small proportion of studies reported the number of patients with an ISS ≥ 15 in each group or the median ISS value. Reported medians ranged from 5 to 33, indicating considerable variation in trauma severity across studies.

Most studies assessed overall or in-hospital mortality. Nine studies reported 30-day mortality [13, 14, 29, 36, 37, 43–45, 67].

**Fig. 2:**
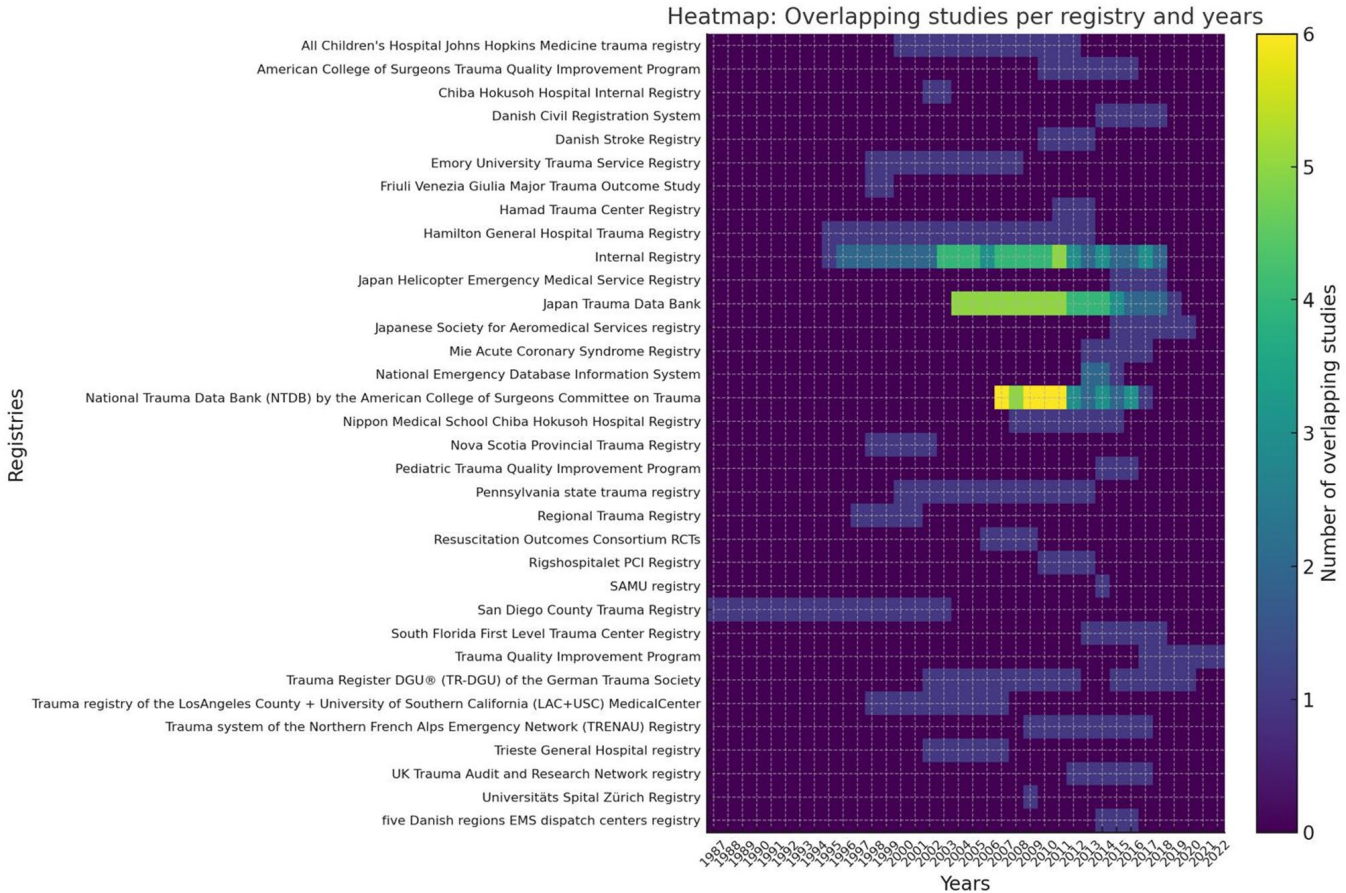
Heatmap of the registers used in the different included studies. The yellow color indicates possible overlap of patients included in some studies that used the same registry and considered the same time frame

The most frequently used case registries were the National Trauma Data Bank (NTDB) of the American College of Surgeons Committee on Trauma and the Japan Trauma Data Bank. In some cases, particularly in retrospective studies, there was potential overlap in the time frames and registries used, because it was not possible to ascertain which patients were included from each registry (Fig. 2).

Fourteen studies evaluated disability outcomes [11, 22, 26, 28, 34–38, 46, 56, 69, 72, 74]. Of these, four focused on trauma patients [11, 38, 56, 74], four on paediatric trauma [22, 26, 34, 35], two on TBI [28, 46], one on stroke [36], one on ACS [37], one on acute aortic dissection [72], and one on mixed populations [69]. Half of the studies were retrospective, and half were prospective or used propensity-score matching.

### Risk of bias (RoB)

In almost all studies, patient selection could have been a potential source of bias. In retrospective studies, the risk of selection bias was high. In propensity-score–matched and prospective studies, there were still concerns about potential confounding due to recruitment through case registries (Figs. 14 S and 15 S in the Supplementary Material).

### Quantitative synthesis for mortality

**Fig. 3:**
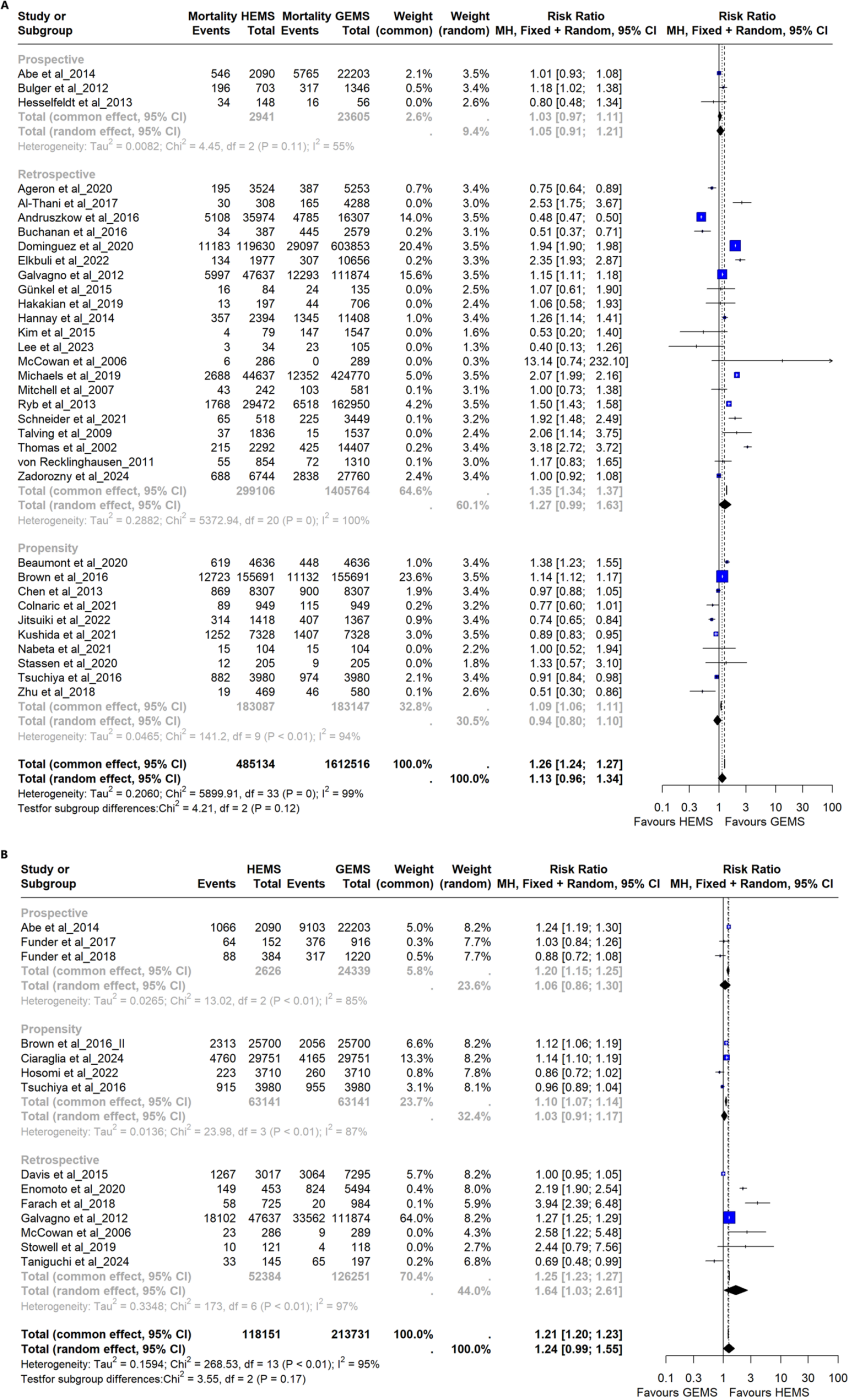
**a**) Forest plots for mortality. The random effects model exhibited an RR of 1.13 (95%CI 0.96–1.34). Since the confidence interval includes the unit, the difference was not statistically significant. We considered only the random-effect model since the high between-study inconsistency was found (99%). **b**) Forest plots for disability. The random effects model exhibited an RR of 1.24 (95%CI 0.99–1.55). Since the confidence interval includes the unit, the difference was not statistically significant. We considered only the random-effect model since the high between-study inconsistency was found (95%)

Across all studies, the relative risk (RR) for mortality in HEMS versus GEMS was 1.13 (95% CI 0.96–1.34; I^2^ = 99%) (Fig. 3). As the confidence interval crossed unity, the result was not statistically significant. In the Bayesian model, HEMS revealed a slightly increased risk compared with GEMS (SMD 0.15; 95%CI 0.03–0.28) (Fig. 9S in the Supplementary Material). After excluding studies derived from the same registry with potential overlapping time frames and retaining only the study with the largest sample size, the random-effects model yielded a RR of 1.16 (95%CI 0.98–1.37, I^2^ = 99%), which, in the same way as the primary analysis, was not statistically significant (Fig. 10S In the Supplementary Material).

**Fig. 4:**
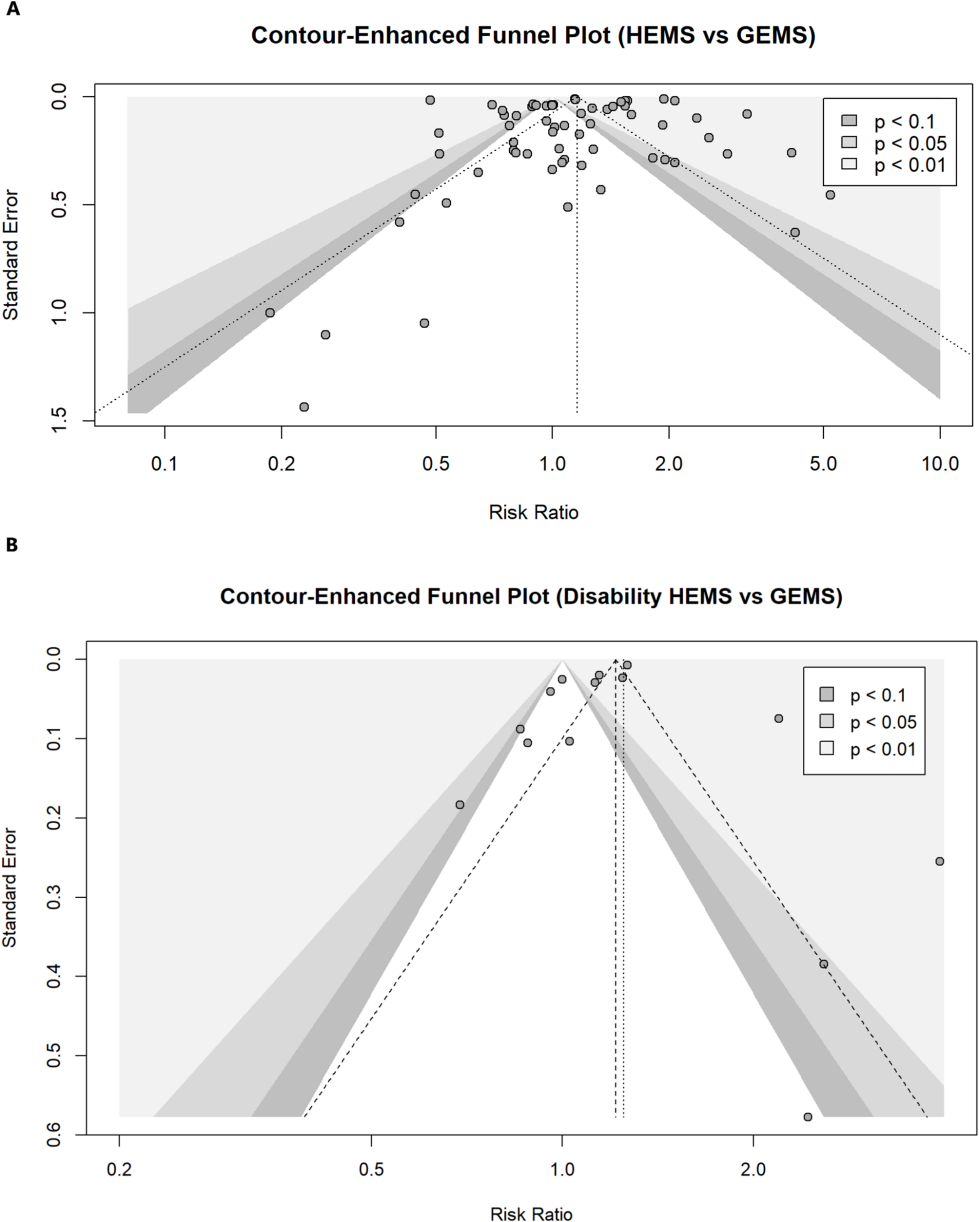
**a**) Funnel plot for mortality outcome. Egger’s test was not statistically significant (p-value = 0.479). **b**) Funnel plot for disability outcome. Egger’s test was not statistically significant (p-value = 0.069)

Funnel plot analysis and the Egger test did not reveal a significant publication bias (*p* = 0.479) (Fig. 4a).

Propensity score matching studies showed that the HEMS group had a RR of 0.94 for mortality (95% CI 0.80–1.10; I^2^ = 94%) compared to the GEMS group (Fig. 3). For prospective studies, the RR was 1.05 (95% CI 0.91–1.21; I^2^ = 55%). Retrospective studies showed a high Inconsistency value: 100% (RR 1.27; 95% CI 0.99–1.63).

**Fig. 5:**
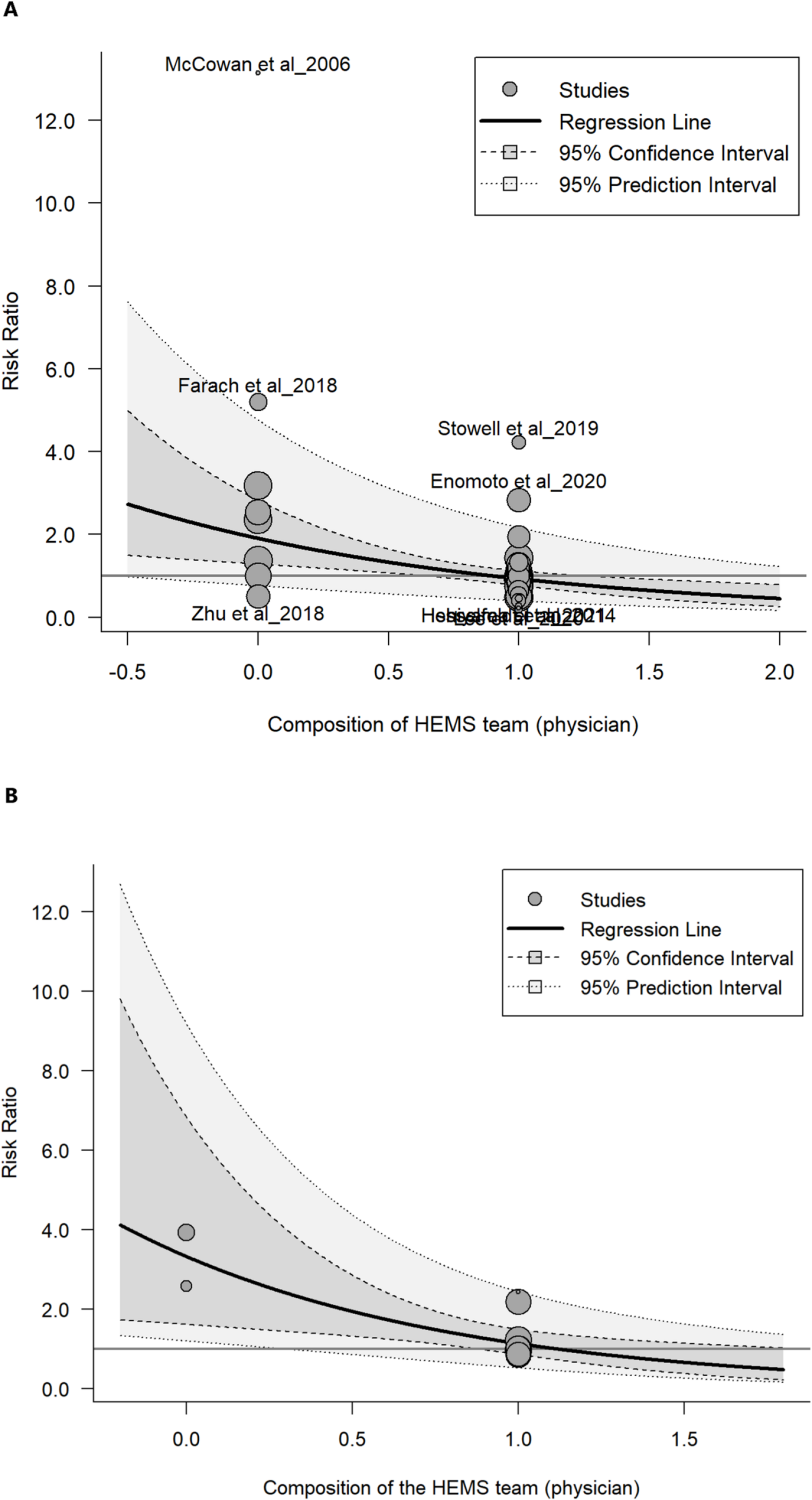
**a**) Meta-regression model for the presence of a physician in the HEMS crew for mortality outcome. The composition of the HEMS crew constitutes about 29% of the heterogeneity. In particular, the presence of a physician leads to a reduction in RR (Est. -0.7230 + 0.2201, *p* = 0.002). **b**) Meta-regression model for the presence of a physician in the HEMS crew for disability outcome. The composition of the HEMS team, in particular the presence of a physician, contribute substantially to heterogeneity (R^2^ = 62%) with an effect which seems favourable to the HEMS group (Est. -1.081 ± 0.344; *p* = 0.0137)

In regression analysis, the study type was responsible for about 17% of heterogeneity, with an increase in mortality in retrospective studies compared to prospective/PSM studies (Est. 0.420 ± 0.138 vs. 0.137 ± 0.220; *p* = 0.003 vs. 0.536). The composition of the HEMS crew accounted for about 29% of the heterogeneity. In particular, the presence of a physician led to a reduction in RR (Est. -0.7230 + 0.2201, *p* = 0.002) (Fig. 5a). When analysing possible sources of heterogeneity through meta-CART model, two moderators were found to be significantly responsible for a difference in effect size: less than 44% of patients with an ISS ≥ 15 in the HEMS group (effect of increasing RR) and the presence of the physician in the HEMS crew (effect of decreasing RR) (Fig. 16S in the Supplementary Material).

Sequential analysis indicated that the effect size approached the alpha boundary for futility, while the sample remained below the beta boundary. This implies that more studies are needed to reach the required information size (Fig. 13S in the Supplementary Material).

In summary, with very low-quality evidence due to confounding risk, wide heterogeneity, and potential multiple imputations from overlapping registries, the overall RR for mortality appeared higher in HEMS than in GEMS (≈ 20 more deaths per 1000 patients), although not statistically significant (Table 2).

**Table 2:** A summary of the GRADE evaluation for assessing the quality of evidence

HEMS compared to GEMS for emergency traumatic and medical conditions						
Patient or population: patients with emergency traumatic and medical conditions Settings: pre-hospital Intervention: HEMS Comparison GEMS						
Outcomes	Illustrative comparative risks* (95% CI)		Relative effect (95% CI)	No of Participants (studies)	Quality of the evidence (GRADE)	Comments
	Assumed risk	Corresponding risk				
	**GEMS**	**HEMS**				
**Mortality** relative risk	**124 per 1000**	**144 per 1000** (127 to 162)	**RR 1.16** (1.02 to 1.31)	1,625,149 (67 studies)	⊕⊝⊝⊝ **very low**	¹ Downgraded for serious risk of bias due to the observational nature of most studies and potential residual confounding. ² Downgraded for inconsistency due to high heterogeneity across studies. ³ Downgraded for indirectness due to variability in patient populations, outcomes definitions, and EMS crew composition.
**Disability** relative risk	**256 per 1000**	**318 per 1000** (254 to 397)	**RR 1.24** (0.99 to 1.55)	331,882 (14 studies)	⊕⊝⊝⊝ **very low**	¹ Downgraded for serious risk of bias due to the observational nature of most studies and potential residual confounding. ² Downgraded for inconsistency due to high heterogeneity across studies. ³ Downgraded for indirectness due to variability in patient populations, outcomes definitions, and EMS crew composition.

*The basis for the **assumed risk** (e.g. the median control group risk across studies) is provided in footnotes. The **corresponding risk** (and its 95% confidence interval) is based on the assumed risk in the comparison group and the **relative effect** of the intervention (and its 95% CI).

**CI**: Confidence interval; **RR**: Risk ratio;

GRADE Working Group grades of evidence

**High quality**: Further research is very unlikely to change our confidence in the estimate of effect.

**Moderate quality**: Further research is likely to have an important impact on our confidence in the estimate of effect and may change the estimate.

**Low quality**: Further research is very likely to have an important impact on our confidence in the estimate of effect and is likely to change the estimate.

**Very low quality**: We are very uncertain about the estimate.

### Subgroup analysis: medical emergencies

There was no significant difference in RR between the HEMS and GEMS groups for the subgroup of patients with ACS (RR 0.77; 95% CI 0.57–1.05; I^2^ = 5%). There was no substantial difference between prospective/PSM studies (Fig. 1S in the Supplementary Material). No statistically significant difference was found for studies conducted on patients with suspected aortic dissection (2 retrospective studies; RR 0.75; 95% CI 0.52–1.07; I^2^ = 0%)(Fig. 2S in the Supplementary Material). No statistically significant difference in RR between the two groups was found for patients with stroke (4 studies; RR 0.91; 95% CI 0.37–2.27; I^2^ = 63%)(Fig. 3S in the Supplementary Material). The only study conducted on septic patients showed an increase in RR for HEMS group patients compared to GEMS group patients (RR 1.81; 95% CI 1.04–3.17)(Fig. 4S in the Supplementary Material).

### Subgroup analysis: pediatric traumatic emergencies

The HEMS group had a higher mortality rate than the GEMS group in studies on pediatric patients with trauma (RR 1.47; 95% CI 1.00–2.18; I^2^ = 97%)(Fig. 5S in the Supplementary Material). The analysis revealed a contrasting result between studies with propensity score matching and retrospective studies (RR 0.79 vs. 1.95, respectively). Neither the composition of the HEMS team (or GEMS team) nor the severity of the trauma explained the high degree of heterogeneity observed.

### Subgroup analysis: TBI

Studies conducted on patients with TBI showed an increased RR for the group of patients treated by HEMS compared with those treated by GEMS (RR 1.21; 95% CI 1.00–1.47; I^2^ = 97%) (Fig. 6S in the Supplementary Material). The only study that used a propensity score matching method revealed, in contrast, a reduction in RR for the HEMS group (RR 0,88; 95% CI 0.81–0.97).

**Fig. 6:**
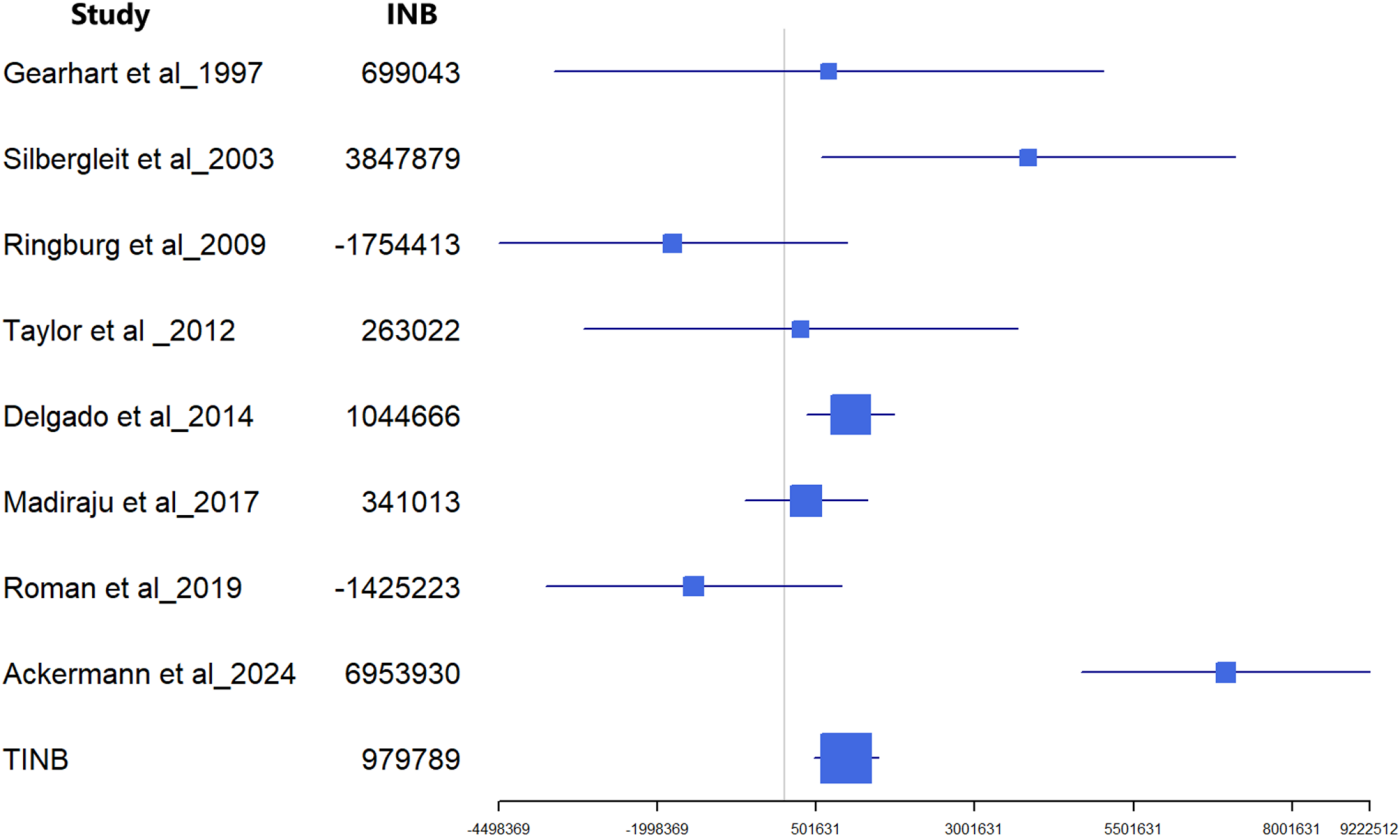
Comparative efficiency research (COMER). The total incremental net benefit (TINB) is roughly 980 thousand euros/QALY/patient, as estimated by the cost-effectiveness studies and the willingness to pay a threshold of 35 million euros for QALY/patient

Considering all studies conducted on patients with TBI, the overall heterogeneity was attributable to the time frame of the records analysed. The years 2007–2014 and 2009–2011 were associated with a statistically significant increase in heterogeneity compared with earlier years (Est 0.45 and 0.44, respectively, *p* = 0.04 for both).

### Subgroup analysis: traumatic patients

For trauma patients, there was no significant difference in RR between those managed by HEMS and those managed by GEMS (RR 1.13; 95% CI 0.96–1.34; I^2^ = 99%). There was also no significant difference in outcomes between prospective, retrospective, or propensity score–matched studies (Fig. 7S in the Supplementary Material).

In the meta-regression, the composition of the HEMS team accounted for about 41% of the heterogeneity between studies (*p* = 0.001). In particular, the presence of a physician was associated with a protective effect on mortality (Est − 0.811 ± 0.246; *p* = 0.0045). The composition of the HEMS team and the number of patients with ISS ≥ 15 in the HEMS group had a synergistic effect, accounting for about 54% of heterogeneity between studies (*p* < 0.001). Similarly, the median ISS value acted synergistically with the composition of the HEMS team, accounting for about 75% of the heterogeneity (*p* < 0.001).

In the influence analysis, several studies appeared to affect the outcome in terms of heterogeneity [11, 12, 15, 16, 22, 23, 25, 27, 30, 32, 38, 49, 53, 57, 65, 66, 73, 74, 76, 77]. Excluding these studies, the remaining 14 studies showed an increased RR for the HEMS group compared with the GEMS group (RR 1.26; 95% CI 1.18–1.34) with low heterogeneity (I^2^ = 39%).

### Subgroup analysis: mixed patients

Three retrospective studies evaluated mixed patients (i.e., both medical and traumatic) and did not show a statistically significant difference in RR for mortality (RR 1.36; 95% CI 0.70–2.64; I^2^ = 64%) (Fig. 8S in the Supplementary Material).

### Quantitative synthesis for disability outcome

Regarding disability outcome, 14 studies evaluated the difference between the two groups. There was no statistically significant difference in RR between the groups (RR 1.24; 95% CI 0.99–1.55; I^2^ = 95%) (Fig. 3b). However, in the sensitivity analysis, after excluding studies with potential overlapping registries and time frames, the RR of disability was significantly higher in patients treated by HEMS compared with those treated by GEMS (RR 1.16; 95% CI 1.02–1.30; I^2^ = 94%) (Fig. 11S in the Supplementary Material).

The Egger test did not detect significant publication bias (*p* = 0.069) (Fig. 4b). The high degree of inconsistency (I^2^ = 95%) should be noted. However, neither the reason for dispatch, nor the type of study (i.e., prospective vs. retrospective), nor the severity of pathology (i.e., ISS for trauma, NIHSS for stroke) contributed significantly to this heterogeneity. By contrast, the composition of the HEMS team, particularly the presence of a physician, contributed substantially to heterogeneity (R^2^ = 62%) with an effect that was favourable to the HEMS group (Est. -1.081 ± 0.344; *p* = 0.0137) (Fig. 5b). In the meta-CART model, the absence of a physician in the HEMS crew was found to be significantly (*p* < 1 × 10^− 4^) associated with a difference in effect size (Fig. 17S in the Supplementary Material).

### Comparative efficiency research (COMER)

The total incremental net benefit (TINB) was roughly € 980,000 per QALY per patient, as estimated by cost-effectiveness studies and using a willingness-to-pay threshold of € 35 million per QALY per patient, based on an economic report from the Lombardy region of northern Italy in 2017 [86] (Fig. 6). However, the incremental net benefit (INB) ranged from € -1,754,413 in Ringburg et al. [80] to € 6,953,930 in Ackermann et al. [85].

## Discussion

According to the current meta-analysis, patients managed by HEMS did not experience a significant increase in survival compared with those managed by GEMS. This was particularly true for patients with medical emergencies. Although no major differences were found in mortality or disability between groups, the conclusions were limited by the marked heterogeneity across studies. The heterogeneity was specifically related to the composition of the HEMS teams and differences in trauma severity between groups. The presence of teams with advanced skills in HEMS compared with GEMS appeared to be a major factor underlying this heterogeneity. HEMS also showed a higher cost-effectiveness ratio in studies that examined this outcome, despite the absence of significant differences in mortality and disability.

A 2015 Cochrane review by Galvagno and colleagues highlighted the wide range of studies in the literature and their methodological weaknesses [87]. Our meta-analysis confirmed, after 10 years, these limitations: the definition of the HEMS team varied according to the study considered. Although it is not plausible that the label alone explains differences in outcomes, the level of skills, especially in trauma, seemed to play a role in the heterogeneity observed. The presence of a physician in the HEMS crew appeared to be a determining factor, together with trauma severity, for the differences in survival rates. Garner et al., reviewing the available literature until 2004, noted that there had been only one RCT addressing survival outcomes in relation to HEMS crew composition [88]. The presence of a physician in the HEMS crew was associated with approximately 35% lower mortality [89]. However, this study was conducted in 1987, and techniques and technologies (e.g., video laryngoscopes) have changed considerably over the past 40 years. Beyond orotracheal intubation, other procedures such as pneumothorax drainage, recognition and treatment strategies for hypovolemic shock in blunt trauma (e.g., endovascular techniques) and penetrating trauma (e.g., thoracotomy), or the prehospital implementation of neuroprotective measures for patients with TBI, could theoretically account for differences in outcomes [90–92].

Comparing survival rates by transport mode was difficult because patients managed by HEMS generally had more severe illness, as reflected by ISS scores. Care delivered by advanced skilled providers was most likely to benefit patients with severe illness compared with those with less severe illness [93]. However, this remained a theoretical assumption rather than one proven by controlled clinical trials [94].

While weak evidence seemed to favour HEMS over GEMS in trauma scenarios, the controversy was even greater in medical emergencies. Our meta-analysis found no difference in mortality between the two groups, which was consistent with previous meta-analyses on stroke patients [95, 96]. However, Florez-Perdomo et al. found that HEMS patients had less disability than those transported by ambulance [96]. This conclusion, although a secondary outcome in our review, was not consistent with their findings. Unlike the review by Florez-Perdomo et al., we grouped patients with different pathologies, as demonstrated by the high degree of inconsistency. Although it might seem intuitive to assume that air transport leads to faster treatment and shorter rescue times in time-dependent pathologies, this is not always the case [97]. In this respect, two considerations should be made: the first concerns the geographical features of the area and the logistical limitations of the EMS setting. The nature of the territory (such as islands or mountainous regions) indicates the need for air transport regardless of performance in terms of target times [98]. The second consideration is that the correlation between rescue time and outcome is not always linear [99]. A longer on-scene duration might suggest more advanced treatment and, consequently, more effective patient management [100].

An additional layer of complexity lay in the interplay between crew composition and total prehospital time. As highlighted by Alstrup et al. [14], the presence of a physician in HEMS teams was associated with improved outcomes and might have allowed for the delivery of more advanced prehospital interventions. Although helicopter transport is often assumed to reduce the time to definitive care, several studies in our review suggested that total prehospital time was actually longer in the HEMS group, largely due to extended on-scene times. However, this additional time was not inherently detrimental; rather, it reflected the opportunity to perform critical procedures such as airway management, haemorrhage control, or ultrasound-guided triage – interventions less frequently available to standard GEMS crews. Conversely, GEMS systems, which vary widely in staffing models, often prioritised rapid transport over on-site stabilisation, potentially at the cost of delayed treatment. Therefore, shorter transport times do not necessarily imply better outcomes, especially when prehospital care is limited in scope.

In our meta-regression and meta-CART analyses, the presence of a physician in the HEMS crew emerged as a significant moderator for both mortality and disability outcomes, suggesting that the quality and intensity of prehospital care might have played a more crucial role than time metrics alone. Future studies should aim to collect granular data on prehospital time intervals and interventions performed, to disentangle the relative contributions of time and team composition to patient outcomes.

Given the nuanced evidence and the considerable investments required to maintain and operate HEMS, future research should aim to refine the criteria for HEMS deployment to ensure its most effective use. Willingness to spend specific amounts may fluctuate over time, which could affect decisions on maintaining a service whose impact is difficult to assess [101]. Enhancing the integration of HEMS into broader emergency care networks, as highlighted by Ageron et al. [12], could maximise its benefits and cost-effectiveness, particularly in regions where geographical barriers substantially affect GEMS response times. In this respect, careful patient selection based on dispatch criteria could help to avoid overuse of a vehicle and rescue crew that might have a greater impact in certain circumstances, while also reducing operating costs.

Establishing the costs of HEMS compared with GEMS remained a challenge. Uncertainty about the benefit of HEMS in relation to GEMS made it difficult to determine whether the cost-effectiveness ratio was advantageous. Cost-effectiveness analyses could only be performed on disability outcomes, since the benefits of HEMS appeared to diminish in terms of mortality. Two additional explanations accounted for these difficulties. First, the studies that conducted such analyses were published over a wide time span: the study by Gearhart et al. was published in 1997, whereas that by Ackermann et al. was published in 2024 [78, 85]. Over the past 27 years, medical knowledge and available technologies have changed considerably. The epidemiology of health emergencies has also evolved, with a global decline in road traffic accidents and an increase in medical emergencies, partly related to the ageing population in industrialised countries [102, 103]. However, the differences in cost and willingness-to-pay thresholds were substantial. The study by Ringburg et al., although not showing a substantial difference between groups in terms of survival or quality of life, concluded that HEMS was affordable [80]. By contrast, the study by Ackermann et al. highlighted that the major expense of HEMS services lay in operating costs, given its 24-hour, seven-day-a-week availability [85]. The results were further influenced by the study of Delgado et al., which also included indirect costs associated with long-term disability [82]. In these terms, HEMS might appear to be an expensive service but was, in fact, cost-effective.

Given the uncertainty of effectiveness compared with less expensive systems, randomised controlled trials are both necessary and justified.

### Limitations

Our review was subject to several limitations, including the diversity of emergency systems worldwide. In our meta-analysis, we included studies based on large trauma registries, acknowledging the possibility of overlapping patient populations due to shared data sources and timeframes. This overlap – particularly among studies using databases such as the Japan Trauma Data Bank or the US National Trauma Data Bank – might have introduced bias in outcome reporting and complicated interpretation of HEMS versus GEMS. Nonetheless, we included all eligible studies to provide a comprehensive overview, considering that excluding studies for potential duplication – without access to patient-level data – might have introduced other forms of bias and reduced the generalisability of the findings. This issue was more pronounced among retrospective studies, which exhibited greater heterogeneity and a trend toward higher reported mortality in HEMS groups compared with prospective or propensity-matched designs. These differences suggested that study design played a meaningful role in shaping the observed effects, contributing to variability in results and highlighting the need for cautious interpretation of retrospective data.

Furthermore, patient outcomes were influenced by the ability of HEMS to offer advanced medical interventions onboard. For example, studies by Alstrup et al. and Al-Thani et al., included in our meta-analysis, described how HEMS teams equipped with specialised medical personnel and equipment could perform life-saving interventions during flight [14, 15]. Although several systems offered both HEMS and GEMS – for instance, most of the prehospital critical care network in the United Kingdom, where the same crew (with identical capabilities) could be deployed by helicopter or car – other networks deployed helicopters and medicalised ground vehicles separately, with two crews of differing skill sets and equipment. GEMS equipment might have shown greater variability (e.g., physician only, physician plus paramedic, paramedic only, nurses, or even volunteers with minimal training). By contrast, provision of HEMS generally required a more consistent minimum level of expertise. This difference could have resulted in differential survival rates in certain patient populations, such as those with severe trauma, for whom time to definitive care was crucial.

The integration of such advanced capabilities in HEMS highlighted discrepancies in outcome studies, which often did not account for variance in prehospital care procedures between HEMS and GEMS. The study by Alstrup et al. underscored the need to consider these capabilities when evaluating the effectiveness and cost-effectiveness of HEMS, suggesting that merely comparing transport times or overall outcomes might have overlooked important benefits conferred by these advanced interventions [14].

## Conclusions

Very low-quality evidence, due to high heterogeneity, confounding from registry-based enrolment, and potential multiple imputation bias, suggested that HEMS did not improve survival compared with GEMS. The presence of teams with advanced skills appeared to confer a modest advantage in reducing disability, particularly in traumatic pathologies. The uncertain outcomes and very low-quality evidence highlight the urgent need for randomised controlled trials with clear definitions of team composition and deployment criteria.

## Supplementary Information

Below is the link to the electronic supplementary material.

Supplementary Material 1

## Data Availability

Data available on reasoned request.
