# Supplementary material for: Survival and cost-effectiveness of helicopter versus ground emergency medical services: a systematic review and meta-analysis with meta-regression and trial sequential analysis
Source: Scand J Trauma Resusc Emerg Med. 2025 Oct 3;33:160. doi: 10.1186/s13049-025-01478-0 (PMC12495656; doi:10.1186/s13049-025-01478-0)

Supplementary Material

- **Table 1S.** Search strategy.
- **Table 2S.** Agreement between the reviewers for titles and abstracts screening and for full-text selection.
- **Figure 1S.** Forest plot for mortality in the ACS patient group. The fixed-effect model exhibited a RR of 0.77 (95%CI 0.57–1.05). Since the confidence interval includes the unit, the difference was not statistically significant.
- **Figure 2S.** Forest plot for mortality in the group of patients with suspected aortic dissection. The fixed-effect model exhibited a RR of 0.75 (95%CI 0.52 – 1.07). Since the confidence interval includes the unit, the difference was not statistically significant.
- **Figure 3S.** Forest plot for mortality in the group of patients with stroke. The random-effects model showed a RR of 0.91 (95%CI 0.37 – 2.27). Since the confidence interval includes the unit, the difference was not statistically significant.
- **Figure 4S.** Forest plot for mortality in the only study on septic patients. The model showed a RR of 1.81 (95%CI 1.04 – 3.17).
- **Figure 5S**. Forest plot for mortality in the group of pediatric patients with trauma. The random-effects model showed a RR of 1.47 (95%CI 1.00 – 2.18). The difference was statistically significant in favor of GEMS.
- **Figure 6S**. Forest plot for mortality in the group of patients with traumatic brain injury. The random-effects model showed a RR of 1.21 (95%CI 1.00 – 1.47). The difference was statistically significant in favor of GEMS.
- **Figure 7S.** Forest plot for mortality in the group of patients with trauma. The random-effects model showed a RR of 1.13 (95%CI 0.96 – 1.34). Since the confidence interval includes the unit, the difference was not statistically significant.
- **Figure 8S.** Forest plot for mortality in the group of mixed patients. The random-effects model showed a RR of 1.36 (95%CI 0.70 – 2.64). Since the confidence interval includes the unit, the difference was not statistically significant.
- **Figure 9S.** Sensitivity analysis for mortality by Bayesian hierarchical model. The SMD was 0.15 (95%CI 0.03 - 0.28). The difference was statistically significant in favor of GEMS.
- **Figure 10S.** Sensitivity analysis for mortality. Forest plot of relative risks (RR) for mortality comparing HEMS versus GEMS. The analysis excludes studies derived from the same registry and overlapping time frames, retaining only the study with the largest sample size. Each horizontal line represents the RR with 95% CI for an individual study; the diamond indicates the pooled estimate. Between-study heterogeneity (I^2) is reported.
- **Figure 11S.** Sensitivity analysis for disability. Forest plot of relative risks (RR) for disability comparing HEMS versus GEMS. The analysis excludes studies derived from the same registry and overlapping time frames, retaining only the study with the largest sample size. Individual study estimates with 95% CIs are shown; the diamond denotes the pooled RR. Between-study heterogeneity (I^2) is indicated.
- **Figure 12S.** P-curve for mortality: 91% of the studies revealed a p-value of 0.01, the remaining 9% reported a p-value of 0.02.
- **Figure 13S.** Trial Sequential Analysis: the cumulative Z-score for the pooled sample size approached the alpha-boundary for futility. However, it did not seem to exceed it. Also did not reach the beta-boundary for 99% statistical power.
- **Figure 14S.** Risk of Bias for prospective and propensity score-matching studies.
- **Figure 15S.** Risk of Bias for retrospective studies.

- **Figure 16S.** Meta-CART model for mortality outcome. Analyzing possible sources of heterogeneity through classification and regression tree (CART) model, two moderators were found to be significantly (p-value =0.0005) responsible for a difference in effect size: less than 44% of patients with a ISS≥15 in the HEMS group (effect of increasing RR) and the presence of the physician in the HEMS crew (effect of decreasing RR).
- **Figure 17S.** Meta-CART model for disability outcome. Analyzing possible sources of heterogeneity through classification and regression tree (CART) model, the absence of the physician in the HEMS crew was found to be significantly (p-value < 10e-4) responsible for a difference in effect size.

Table 1S

Library	Query	Records
PubMed	((“HEMS” OR “helicopter emergency medical system” OR “air emergency medical system”) AND (“GEMS” OR “ground emergency medical system” OR “ambulance emergency medical system” OR “car emergency medical system”)) NOT (animal[mh] NOT human[mh]) NOT (comment[pt] OR editorial[pt] OR meta-analysis[pt] OR practice-guideline[pt] OR review[pt]))	756
Scopus	(TITLE-ABS-KEY (helicopter AND emergency medical system) AND TITLE-ABS-KEY (HEMS AND GEMS) OR TITLE-ABS-KEY (HEMS AND helicopter AND emergency AND system) OR TITLE-ABS-KEY (HEMS) OR TITLE-ABS-KEY (HEMS AND mortality) AND NOT TITLE-ABS-KEY (case AND report) AND NOT TITLE-ABS-KEY (review AND article) AND NOT TITLE-ABS-KEY (animal AND model) AND NOT TITLE-ABS-KEY (review AND literature) AND NOT TITLE-ABS-KEY (systematic AND review))	116
CINHAL	(TITLE-ABS-KEY (helicopter AND emergency medical system) AND TITLE-ABS-KEY (HEMS AND GEMS) OR TITLE-ABS-KEY (HEMS AND helicopter AND emergency AND system) OR TITLE-ABS-KEY (HEMS) OR TITLE-ABS-KEY (HEMS AND mortality) AND NOT TITLE-ABS-KEY (case AND report) AND NOT TITLE-ABS-KEY (review AND article) AND NOT TITLE-ABS-KEY (animal AND model) AND NOT TITLE-ABS-KEY (review AND literature) AND NOT TITLE-ABS-KEY (systematic AND review))	212
Web of Science	(TITLE-ABS-KEY (helicopter AND emergency medical system) AND TITLE-ABS-KEY (HEMS AND GEMS) OR TITLE-ABS-KEY (HEMS AND helicopter AND emergency AND system) OR TITLE-ABS-KEY (HEMS) OR TITLE-ABS-KEY (HEMS AND mortality) AND NOT TITLE-ABS-KEY (case AND report) AND NOT TITLE-ABS-KEY (review AND article) AND NOT TITLE-ABS-KEY (animal AND model) AND NOT TITLE-ABS-KEY (review AND literature) AND NOT TITLE-ABS-KEY (systematic AND review))	511

Table 2S

Titles and Abstracts screening					
Pairwise comparison	Proportionate Agreement	Yes Probability	No Probability	Random Agreement Probability	Cohen's Kappa
DO vs TB	0.88416	0.09045	0.48761	0.57806	0.72546
DO vs UGS	0.88889	0.05564	0.57416	0.62979	0.69987
TB vs UGS	0.78601	0.04954	0.5804	0.62993	0.42175
Full text selection					
Pairwise comparison	Proportionate Agreement	Yes Probability	No Probability	Random Agreement Probability	Cohen's Kappa
DO vs LF	0.76923	0.53254	0.07101	0.60355	0.41791
DO vs TB	0.86905	0.23158	0.26729	0.49887	0.73869

Figure 1S

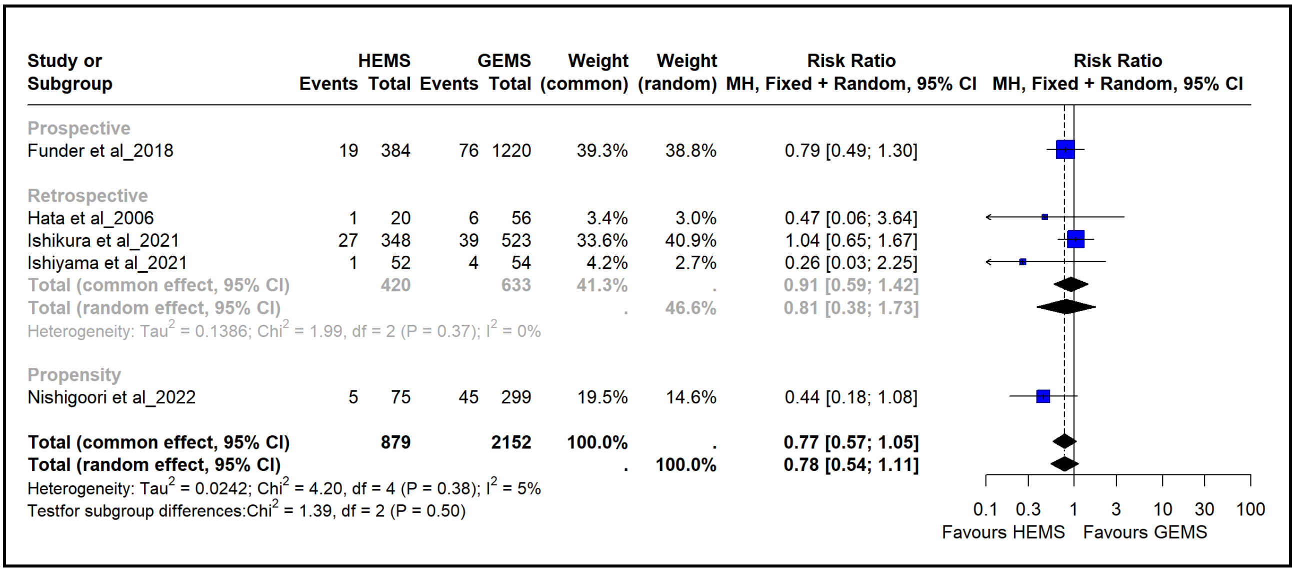

Figure 2S

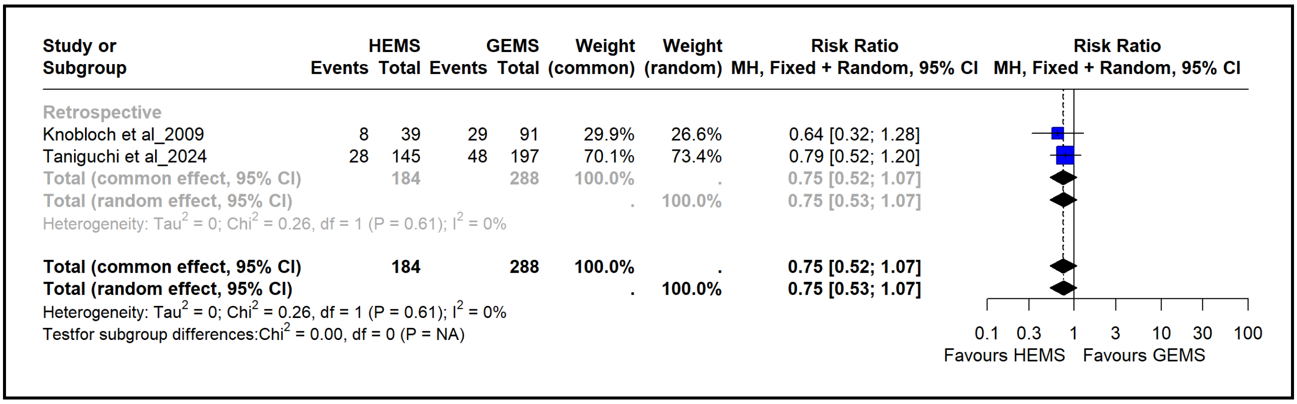

Figure 3S

Study or Subgroup	HEMS Events	HEMS Total	GEMS Events	GEMS Total	Weight (common)	Weight (random)	Risk Ratio MH, Fixed + Random, 95% CI
Prospective							
Funder et al_2017	46	152	258	916	76.1%	40.9%	1.07 [0.83; 1.40]
Hesselfeldt et al_2014	0	22	8	87	3.7%	8.4%	0.23 [0.01; 3.82]
Total (common effect, 95% CI)		174		1003	79.8%	.	1.04 [0.80; 1.35]
Total (random effect, 95% CI)					.	49.3%	0.96 [0.44; 2.11]
Heterogeneity: $Tau^2 = 0.1566$; $Chi^2 = 1.15$, df = 1 (P = 0.28); $I^2 = 13\%$							
Retrospective							
Lee et al_2020	1	26	21	102	8.8%	14.3%	0.19 [0.03; 1.33]
Ueno et al_2019	12	61	49	485	11.4%	36.3%	1.95 [1.10; 3.45]
Total (common effect, 95% CI)		87		587	20.2%	.	1.18 [0.69; 2.01]
Total (random effect, 95% CI)					.	50.7%	0.73 [0.08; 7.06]
Heterogeneity: $Tau^2 = 2.2049$; $Chi^2 = 5.07$, df = 1 (P = 0.02); $I^2 = 80\%$							
**Total (common effect, 95% CI)**		**261**		**1590**	**100.0%**	**.**	**1.06 [0.84; 1.35]**
**Total (random effect, 95% CI)**					**.**	**100.0%**	**0.91 [0.37; 2.27]**
Heterogeneity: $Tau^2 = 0.5117$; $Chi^2 = 8.08$, df = 3 (P = 0.04); $I^2 = 63\%$							
Testfor subgroup differences: $Chi^2 = 0.05$, df = 1 (P = 0.83)							

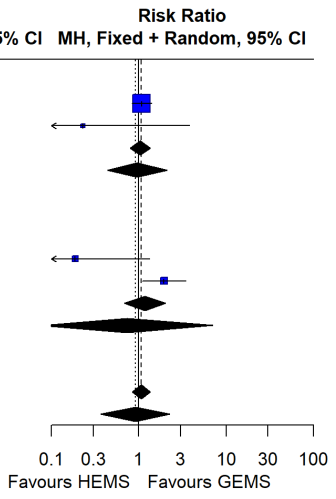

Figure 4S

Study or Subgroup	HEMS Events	HEMS Total	GEMS Events	GEMS Total	Risk Ratio MH, 95% CI
**Retrospective**					
Kashyap et al_2016	18	60	20	121	1.81 [1.04; 3.17]

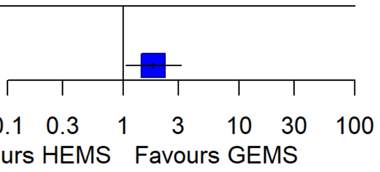

Figure 5S

Study or Subgroup	Mortality HEMS Events	Mortality HEMS Total	Mortality GEMS Events	Mortality GEMS Total	Weight (common)	Weight (random)	Risk Ratio MH, Fixed + Random, 95% CI
**Retrospective**							
Bläsius et al_2021	65	826	150	1929	2.3%	10.3%	1.01 [0.77; 1.34]
Duffens et al_2022	90	699	143	1391	2.4%	10.4%	1.25 [0.98; 1.60]
Englum et al_2017	781	10565	1287	26808	18.3%	10.8%	1.54 [1.41; 1.68]
Enomoto et al_2020	17	453	73	5494	0.3%	9.1%	2.82 [1.68; 4.75]
Farach et al_2018	23	725	6	984	0.1%	6.9%	5.20 [2.13; 12.71]
Missios et al_2014	194	2445	440	8864	4.8%	10.7%	1.60 [1.36; 1.88]
Stewart et al_2015	82	779	17	667	0.5%	9.2%	4.13 [2.48; 6.89]
Total (common effect, 95% CI)		16492		46137	28.6%	.	1.55 [1.45; 1.66]
Total (random effect, 95% CI)					.	67.6%	1.95 [1.28; 2.96]
Heterogeneity: $Tau^2 = 0.2725$; $Chi^2 = 38.19$, df = 6 ($P < 0.01$); $I^2 = 84\%$							
**Propensity**							
Brown et al_2016_II	1096	25700	1570	25700	39.5%	10.9%	0.70 [0.65; 0.75]
Ciaraglia et al_2024	909	29751	1020	29751	25.6%	10.8%	0.89 [0.82; 0.97]
Polites et al_2017	202	2242	250	2242	6.3%	10.7%	0.81 [0.68; 0.96]
Total (common effect, 95% CI)		57693		57693	71.4%	.	0.78 [0.74; 0.82]
Total (random effect, 95% CI)					.	32.4%	0.79 [0.68; 0.92]
Heterogeneity: $Tau^2 = 0.0150$; $Chi^2 = 17.32$, df = 2 ($P < 0.01$); $I^2 = 88\%$							
**Total (common effect, 95% CI)**		**74185**		**103830**	**100.0%**	**.**	**1.00 [0.96; 1.04]**
**Total (random effect, 95% CI)**					**.**	**100.0%**	**1.47 [1.00; 2.18]**

Heterogeneity: $Tau^2 = 0.3649$; $Chi^2 = 289.53$, df = 9 ($P < 0.01$); $I^2 = 97\%$

Testfor subgroup differences:$Chi^2 = 15.70$, df = 1 ($P < 0.01$)

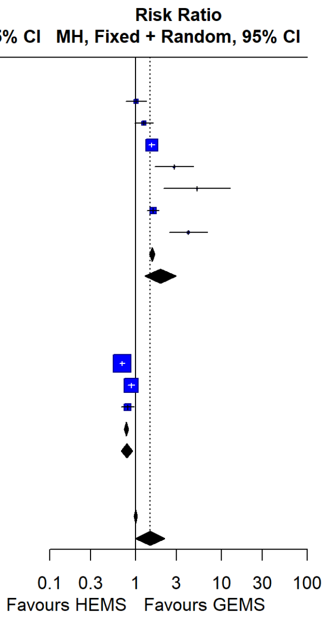

Figure 6S

Study or Subgroup	Mortality HEMS Events	Mortality HEMS Total	Mortality GEMS Events	Mortality GEMS Total	Weight (common)	Weight (random)	Risk Ratio MH, Fixed + Random, 95% CI
Retrospective							
Aiolfi et al_2018	3468	29168	8872	116391	39.6%	16.5%	1.56 [1.50; 1.62]
Bekelis et al_2015	2797	23283	8161	104548	33.1%	16.5%	1.54 [1.48; 1.60]
Berlot et al_2009	19	89	26	105	0.3%	7.5%	0.86 [0.51; 1.45]
Davis et al_2015	759	3017	1845	7295	12.0%	16.2%	0.99 [0.92; 1.07]
Ota et al_2021	406	2029	5506	39329	6.0%	16.0%	1.43 [1.31; 1.56]
Sun et al_2017	6	360	10	658	0.1%	3.0%	1.10 [0.40; 2.99]
Total (common effect, 95% CI)		57946		268326	91.0%	.	1.47 [1.43; 1.50]
Total (random effect, 95% CI)					.	75.8%	1.30 [1.06; 1.58]
Heterogeneity: $Tau^2 = 0.0461$; $Chi^2 = 129$, df = 5 ($P < 0.01$); $I^2 = 96\%$							
Prospective							
Di Bartolomeo et al_2001	28	92	22	92	0.2%	8.2%	1.27 [0.79; 2.05]
Propensity							
Hosomi et al_2022	696	3710	787	3710	8.8%	16.0%	0.88 [0.81; 0.97]
**Total (common effect, 95% CI)**		**61748**		**272128**	**100.0%**	**.**	**1.42 [1.38; 1.45]**
**Total (random effect, 95% CI)**					**.**	**100.0%**	**1.21 [1.00; 1.47]**
Heterogeneity: $Tau^2 = 0.0580$; $Chi^2 = 238.14$, df = 7 ($P < 0.01$); $I^2 = 97\%$							
Testfor subgroup differences:$Chi^2 = 13.18$, df = 2 ($P < 0.01$)							

Risk Ratio
MH, Fixed + Random, 95% CI

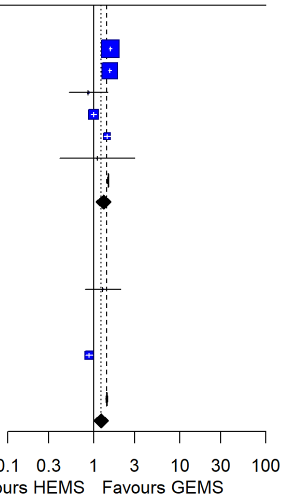

Figure 7S

Study or Subgroup	Mortality HEMS Events	Mortality HEMS Total	Mortality GEMS Events	Mortality GEMS Total	Weight (common)	Weight (random)	Risk Ratio MH, Fixed + Random, 95% CI
Prospective							
Abe et al_2014	546	2090	5765	22203	2.1%	3.5%	1.01 [0.93; 1.08]
Bulger et al_2012	196	703	317	1346	0.5%	3.4%	1.18 [1.02; 1.38]
Hesselfeldt et al_2013	34	148	16	56	0.0%	2.6%	0.80 [0.48; 1.34]
Total (common effect, 95% CI)		2941		23605	2.6%	.	1.03 [0.97; 1.11]
Total (random effect, 95% CI)					.	9.4%	1.05 [0.91; 1.21]
Heterogeneity: Tau^2 = 0.0082; Chi^2 = 4.45, df = 2 (P = 0.11); I^2 = 55%							
Retrospective							
Ageron et al_2020	195	3524	387	5253	0.7%	3.4%	0.75 [0.64; 0.89]
Al-Thani et al_2017	30	308	165	4288	0.0%	3.0%	2.53 [1.75; 3.67]
Andruszkow et al_2016	5108	35974	4785	16307	14.0%	3.5%	0.48 [0.47; 0.50]
Buchanan et al_2016	34	387	445	2579	0.2%	3.1%	0.51 [0.37; 0.71]
Dominguez et al_2020	11183	119630	29097	603853	20.4%	3.5%	1.94 [1.90; 1.98]
Elkbuli et al_2022	134	1977	307	10656	0.2%	3.3%	2.35 [1.93; 2.87]
Galvagno et al_2012	5997	47637	12293	111874	15.6%	3.5%	1.15 [1.11; 1.18]
Günkel et al_2015	16	84	24	135	0.0%	2.5%	1.07 [0.61; 1.90]
Hakakian et al_2019	13	197	44	706	0.0%	2.4%	1.06 [0.58; 1.93]
Hannay et al_2014	357	2394	1345	11408	1.0%	3.4%	1.26 [1.14; 1.41]
Kim et al_2015	4	79	147	1547	0.0%	1.6%	0.53 [0.20; 1.40]
Lee et al_2023	3	34	23	105	0.0%	1.3%	0.40 [0.13; 1.26]
McCowan et al_2006	6	286	0	289	0.0%	0.3%	13.14 [0.74; 232.10]
Michaels et al_2019	2688	44637	12352	424770	5.0%	3.5%	2.07 [1.99; 2.16]
Mitchell et al_2007	43	242	103	581	0.1%	3.1%	1.00 [0.73; 1.38]
Ryb et al_2013	1768	29472	6518	162950	4.2%	3.5%	1.50 [1.43; 1.58]
Schneider et al_2021	65	518	225	3449	0.1%	3.2%	1.92 [1.48; 2.49]
Talving et al_2009	37	1836	15	1537	0.0%	2.4%	2.06 [1.14; 3.75]
Thomas et al_2002	215	2292	425	14407	0.2%	3.4%	3.18 [2.72; 3.72]
von Recklinghausen_2011	55	854	72	1310	0.1%	3.0%	1.17 [0.83; 1.65]
Zadorozny et al_2024	688	6744	2838	27760	2.4%	3.4%	1.00 [0.92; 1.08]
Total (common effect, 95% CI)		299106		1405764	64.6%	.	1.35 [1.34; 1.37]
Total (random effect, 95% CI)					.	60.1%	1.27 [0.99; 1.63]
Heterogeneity: Tau^2 = 0.2882; Chi^2 = 5372.94, df = 20 (P = 0); I^2 = 100%							
Propensity							
Beaumont et al_2020	619	4636	448	4636	1.0%	3.4%	1.38 [1.23; 1.55]
Brown et al_2016	12723	155691	11132	155691	23.6%	3.5%	1.14 [1.12; 1.17]
Chen et al_2013	869	8307	900	8307	1.9%	3.4%	0.97 [0.88; 1.05]
Colnaric et al_2021	89	949	115	949	0.2%	3.2%	0.77 [0.60; 1.01]
Jitsuiki et al_2022	314	1418	407	1367	0.9%	3.4%	0.74 [0.65; 0.84]
Kushida et al_2021	1252	7328	1407	7328	3.0%	3.5%	0.89 [0.83; 0.95]
Nabeta et al_2021	15	104	15	104	0.0%	2.2%	1.00 [0.52; 1.94]
Stassen et al_2020	12	205	9	205	0.0%	1.8%	1.33 [0.57; 3.10]
Tsuchiya et al_2016	882	3980	974	3980	2.1%	3.4%	0.91 [0.84; 0.98]
Zhu et al_2018	19	469	46	580	0.1%	2.6%	0.51 [0.30; 0.86]
Total (common effect, 95% CI)		183087		183147	32.8%	.	1.09 [1.06; 1.11]
Total (random effect, 95% CI)					.	30.5%	0.94 [0.80; 1.10]
Heterogeneity: Tau^2 = 0.0465; Chi^2 = 141.2, df = 9 (P < 0.01); I^2 = 94%							
**Total (common effect, 95% CI)**		**485134**		**1612516**	**100.0%**	**.**	**1.26 [1.24; 1.27]**
**Total (random effect, 95% CI)**					**.**	**100.0%**	**1.13 [0.96; 1.34]**

Heterogeneity: Tau^2 = 0.2060; Chi^2 = 5899.91, df = 33 (P = 0); I^2 = 99%
Testfor subgroup differences:Chi^2 = 4.21, df = 2 (P = 0.12)

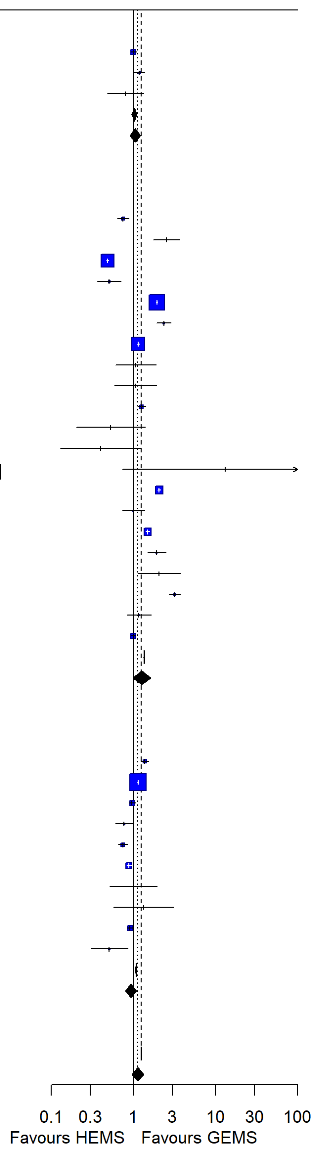

Figure 8S

Study or Subgroup	HEMS Events	HEMS Total	GEMS Events	GEMS Total	Weight (common)	Weight (random)	Risk Ratio MH, Fixed + Random, 95% CI
Retrospective							
Alsrtup et al_2021	492	2260	71	315	86.1%	47.2%	0.97 [0.78; 1.20]
Herrlin Jensen et al_2021	22	318	16	275	11.8%	34.5%	1.19 [0.64; 2.22]
Stowell et al_2019	13	121	3	118	2.1%	18.3%	4.23 [1.24; 14.45]
Total (common effect, 95% CI)		2699		708	100.0%	.	1.06 [0.87; 1.30]
Total (random effect, 95% CI)					.	100.0%	1.36 [0.70; 2.64]
Heterogeneity: Tau^2 = 0.2297; Chi^2 = 5.6, df = 2 (P = 0.06); I^2 = 64%							
**Total (common effect, 95% CI)**		**2699**		**708**	**100.0%**	**.**	**1.06 [0.87; 1.30]**
**Total (random effect, 95% CI)**					**.**	**100.0%**	**1.36 [0.70; 2.64]**

Heterogeneity: Tau^2 = 0.2297; Chi^2 = 5.60, df = 2 (P = 0.06); I^2 = 64%
Testfor subgroup differences:Chi^2 = 0.00, df = 0 (P = NA)

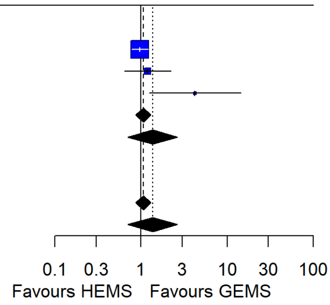

Figure 9S

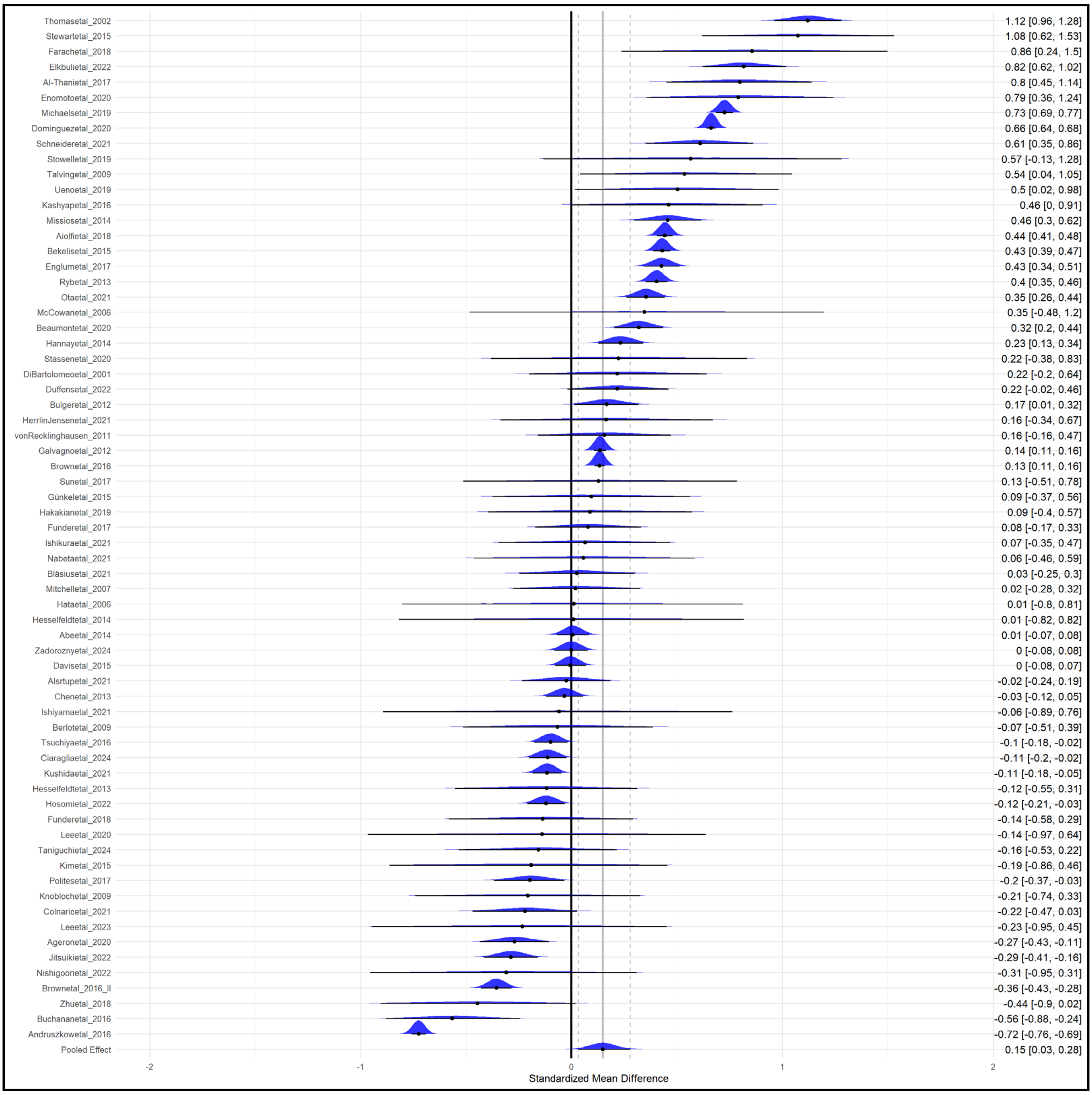
Thomasetal_2002 1.12 [0.96, 1.28]
Stewartetal_2015 1.08 [0.62, 1.53]
Farachetal_2018 0.86 [0.24, 1.5]
Elkbulietal_2022 0.82 [0.62, 1.02]
Al-Thanietal_2017 0.8 [0.45, 1.14]
Enomotoetal_2020 0.79 [0.36, 1.24]
Michaelsetal_2019 0.73 [0.69, 0.77]
Dominguezetal_2020 0.66 [0.64, 0.68]
Schneideretal_2021 0.61 [0.35, 0.86]
Stowelletal_2019 0.57 [-0.13, 1.28]
Talvingetal_2009 0.54 [0.04, 1.05]
Uenoetal_2019 0.5 [0.02, 0.98]
Kashyapetal_2016 0.46 [0, 0.91]
Missiosetal_2014 0.46 [0.3, 0.62]
Aiolfietal_2018 0.44 [0.41, 0.48]
Bekelisetal_2015 0.43 [0.39, 0.47]
Englumetal_2017 0.43 [0.34, 0.51]
Rybetal_2013 0.4 [0.35, 0.46]
Otaetal_2021 0.35 [0.26, 0.44]
McCowanetal_2006 0.35 [-0.48, 1.2]
Beaumontetal_2020 0.32 [0.2, 0.44]
Hannayetal_2014 0.23 [0.13, 0.34]
Stassenetal_2020 0.22 [-0.38, 0.83]
DiBartolomeoetal_2001 0.22 [-0.2, 0.64]
Duffensetal_2022 0.22 [-0.02, 0.46]
Bulgeretal_2012 0.17 [0.01, 0.32]
HerrlinJensenetal_2021 0.16 [-0.34, 0.67]
vonRecklinghausen_2011 0.16 [-0.16, 0.47]
Galvagnoetal_2012 0.14 [0.11, 0.16]
Brownetal_2016 0.13 [0.11, 0.16]
Sunetal_2017 0.13 [-0.51, 0.78]
Günkeletal_2015 0.09 [-0.37, 0.56]
Hakakianetal_2019 0.09 [-0.4, 0.57]
Funderetal_2017 0.08 [-0.17, 0.33]
Ishikuraetal_2021 0.07 [-0.35, 0.47]
Nabetaetal_2021 0.06 [-0.46, 0.59]
Bläsiusetal_2021 0.03 [-0.25, 0.3]
Mitchelletal_2007 0.02 [-0.28, 0.32]
Hataetal_2006 0.01 [-0.8, 0.81]
Hesselfeldtetal_2014 0.01 [-0.82, 0.82]
Abeetal_2014 0.01 [-0.07, 0.08]
Zadoroznyetal_2024 0 [-0.08, 0.08]
Davisetal_2015 0 [-0.08, 0.07]
Alsrtupetal_2021 -0.02 [-0.24, 0.19]
Chenetal_2013 -0.03 [-0.12, 0.05]
Ishiyamaetal_2021 -0.06 [-0.89, 0.76]
Berlotetal_2009 -0.07 [-0.51, 0.39]
Tsuchiyaetal_2016 -0.1 [-0.18, -0.02]
Ciaragliaetal_2024 -0.11 [-0.2, -0.02]
Kushidaetal_2021 -0.11 [-0.18, -0.05]
Hesselfeldtetal_2013 -0.12 [-0.55, 0.31]
Hosomietal_2022 -0.12 [-0.21, -0.03]
Funderetal_2018 -0.14 [-0.58, 0.29]
Leeetal_2020 -0.14 [-0.97, 0.64]
Taniguchietal_2024 -0.16 [-0.53, 0.22]
Kimetal_2015 -0.19 [-0.86, 0.46]
Politesetal_2017 -0.2 [-0.37, -0.03]
Knoblochetal_2009 -0.21 [-0.74, 0.33]
Colnaricetal_2021 -0.22 [-0.47, 0.03]
Leeetal_2023 -0.23 [-0.95, 0.45]
Ageronetal_2020 -0.27 [-0.43, -0.11]
Jitsuiketal_2022 -0.29 [-0.41, -0.16]
Nishigoorietal_2022 -0.31 [-0.95, 0.31]
Brownetal_2016_II -0.36 [-0.43, -0.28]
Zhuetal_2018 -0.44 [-0.9, 0.02]
Buchananetal_2016 -0.56 [-0.88, -0.24]
Andruszkowetal_2016 -0.72 [-0.76, -0.69]
Pooled Effect 0.15 [0.03, 0.28]
-2
-1
0
1
2
Standardized Mean Difference

Figure 10S

Study	HEMS Events	HEMS HEMS N	GEMS Events	GEMS GEMS N	RR	95%-CI	Weight (common)	Weight (random)
Ageron et al_2020	195	3524	387	5253	0.75	[0.64; 0.89]	0.9%	2.5%
Alsrtup et al_2021	492	2260	71	315	0.97	[0.78; 1.20]	0.3%	2.4%
Al-Thani et al_2017	30	308	165	4288	2.53	[1.75; 3.67]	0.1%	2.2%
Andruszkow et al_2016	5108	35974	4785	16307	0.48	[0.47; 0.50]	18.2%	2.5%
Beaumont et al_2020	619	4636	448	4636	1.38	[1.23; 1.55]	1.2%	2.5%
Berlot et al_2009	19	89	26	105	0.86	[0.51; 1.45]	0.1%	2.0%
Bläsius et al_2021	65	826	150	1929	1.01	[0.77; 1.34]	0.2%	2.3%
Brown et al_2016	12723	155691	11132	155691	1.14	[1.12; 1.17]	30.7%	2.5%
Buchanan et al_2016	34	387	445	2579	0.51	[0.37; 0.71]	0.3%	2.3%
Bulger et al_2012	196	703	317	1346	1.18	[1.02; 1.38]	0.6%	2.5%
Chen et al_2013	869	8307	900	8307	0.97	[0.88; 1.05]	2.5%	2.5%
Colnaric et al_2021	89	949	115	949	0.77	[0.60; 1.01]	0.3%	2.4%
Davis et al_2015	759	3017	1845	7295	0.99	[0.92; 1.07]	3.0%	2.5%
Di Bartolomeo et al_2001	28	92	22	92	1.27	[0.79; 2.05]	0.1%	2.1%
Dominguez et al_2020	11183	119630	29097	603853	1.94	[1.90; 1.98]	26.6%	2.5%
Duffens et al_2022	90	699	143	1391	1.25	[0.98; 1.60]	0.3%	2.4%
Elkbuli et al_2022	134	1977	307	10656	2.35	[1.93; 2.87]	0.3%	2.4%
Farach et al_2018	23	725	6	984	5.20	[2.13; 12.71]	0.0%	1.5%
Funder et al_2017	46	152	258	916	1.07	[0.83; 1.40]	0.2%	2.4%
Funder et al_2018	19	384	76	1220	0.79	[0.49; 1.30]	0.1%	2.1%
Günkel et al_2015	16	84	24	135	1.07	[0.61; 1.90]	0.1%	1.9%
Hakakian et al_2019	13	197	44	706	1.06	[0.58; 1.93]	0.1%	1.9%
Hannay et al_2014	357	2394	1345	11408	1.26	[1.14; 1.41]	1.3%	2.5%
Hata et al_2006	1	20	6	56	0.47	[0.06; 3.64]	0.0%	0.5%
Herrlin Jensen et al_2021	22	318	16	275	1.19	[0.64; 2.22]	0.0%	1.9%
Hesselfeldt et al_2013	34	148	16	56	0.80	[0.48; 1.34]	0.1%	2.0%
Hesselfeldt et al_2014	0	22	8	87	0.23	[0.01; 3.82]	0.0%	0.3%
Ishikura et al_2021	27	348	39	523	1.04	[0.65; 1.67]	0.1%	2.1%
Ishiyama et al_2021	1	52	4	54	0.26	[0.03; 2.25]	0.0%	0.5%
Kashyap et al_2016	18	60	20	121	1.81	[1.04; 3.17]	0.0%	2.0%
Kim et al_2015	4	79	147	1547	0.53	[0.20; 1.40]	0.0%	1.4%
Knobloch et al_2009	8	39	29	91	0.64	[0.32; 1.28]	0.0%	1.8%
Lee et al_2020	1	26	21	102	0.19	[0.03; 1.33]	0.0%	0.6%
Lee et al_2023	3	34	23	105	0.40	[0.13; 1.26]	0.0%	1.2%
McCowan et al_2006	6	286	0	289	13.14	[0.74; 232.10]	0.0%	0.3%
Michaels et al_2019	2688	44637	12352	424770	2.07	[1.99; 2.16]	6.5%	2.5%
Mitchell et al_2007	43	242	103	581	1.00	[0.73; 1.38]	0.2%	2.3%
Nabeta et al_2021	15	104	15	104	1.00	[0.52; 1.94]	0.0%	1.8%
Nishigoori et al_2022	5	75	45	299	0.44	[0.18; 1.08]	0.0%	1.5%
Ota et al_2021	406	2029	5506	39329	1.43	[1.31; 1.56]	1.5%	2.5%
von Recklinghausen_2011	55	854	72	1310	1.17	[0.83; 1.65]	0.2%	2.3%
Schneider et al_2021	65	518	225	3449	1.92	[1.48; 2.49]	0.2%	2.4%
Stassen et al_2020	12	205	9	205	1.33	[0.57; 3.10]	0.0%	1.5%
Stewart et al_2015	82	779	17	667	4.13	[2.48; 6.89]	0.1%	2.0%
Stowell et al_2019	13	121	3	118	4.23	[1.24; 14.45]	0.0%	1.1%
Talving et al_2009	37	1836	15	1537	2.06	[1.14; 3.75]	0.0%	1.9%
Taniguchi et al_2024	28	145	48	197	0.79	[0.52; 1.20]	0.1%	2.2%
Thomas et al_2002	215	2292	425	14407	3.18	[2.72; 3.72]	0.3%	2.5%
Ueno et al_2019	12	61	49	485	1.95	[1.10; 3.45]	0.0%	1.9%
Zadorozny et al_2024	688	6744	2838	27760	1.00	[0.92; 1.08]	3.1%	2.5%
Zhu et al_2018	19	469	46	580	0.51	[0.30; 0.86]	0.1%	2.0%
**Common effect model**		**405548**		**1359465**	**1.30**	**[1.28; 1.31]**	**100.0%**	**.**
**Random effects model**					**1.16**	**[0.98; 1.37]**	**.**	**100.0%**

Heterogeneity: $I^2 = 99.1\%$, $\tau^2 = 0.2868$, $p = 0$

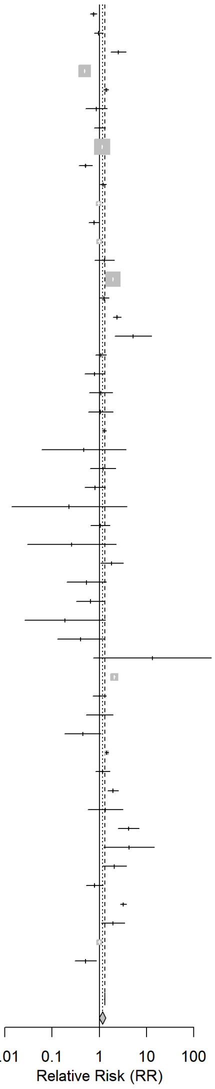

Figure 11S

Study	HEMS Disability	HEMS N	GEMS Disability	GEMS N	RR	95%-CI	Weight (common)	Weight (random)
Abe et al_2014	1066	2090	9103	22203	1.24	[1.19; 1.30]	6.6%	19.6%
Davis et al_2015	1267	3017	3064	7295	1.00	[0.95; 1.05]	7.5%	19.5%
Farach et al_2018	58	725	20	984	3.94	[2.39; 6.48]	0.1%	4.5%
Funder et al_2017	64	152	376	916	1.03	[0.84; 1.26]	0.5%	12.9%
Funder et al_2018	88	384	317	1220	0.88	[0.72; 1.08]	0.6%	12.7%
Galvagno et al_2012	18102	47637	33562	111874	1.27	[1.25; 1.29]	84.4%	20.1%
McCowan et al_2006	23	286	9	289	2.58	[1.22; 5.48]	0.0%	2.3%
Stowell et al_2019	10	121	4	118	2.44	[0.79; 7.56]	0.0%	1.1%
Taniguchi et al_2024	33	145	65	197	0.69	[0.48; 0.99]	0.2%	7.2%
**Common effect model**		**54557**		**145096**	**1.24**	**[1.23; 1.26]**	**100.0%**	**.**
**Random effects model**					**1.16**	**[1.02; 1.30]**	**.**	**100.0%**

Heterogeneity: $I^2 = 93.8\%$, $\tau^2 = 0.0187$, $p < 0.0001$

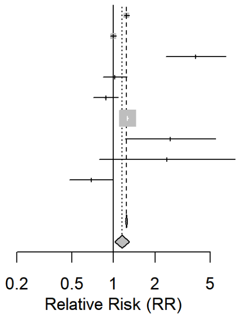

Figure 12S

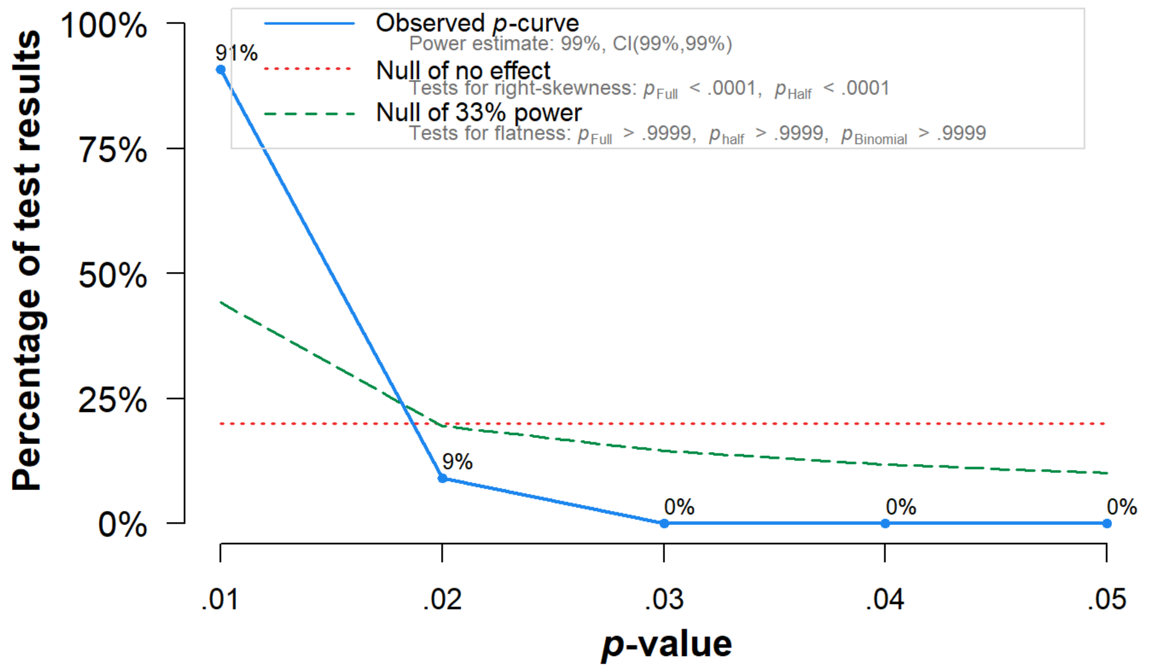

Note: The observed p-curve includes 22 statistically significant ($p < .05$) results, of which 22 are $p < .025$. There were 2 additional results entered but excluded from p-curve because they were $p > .05$.

Figure 13S

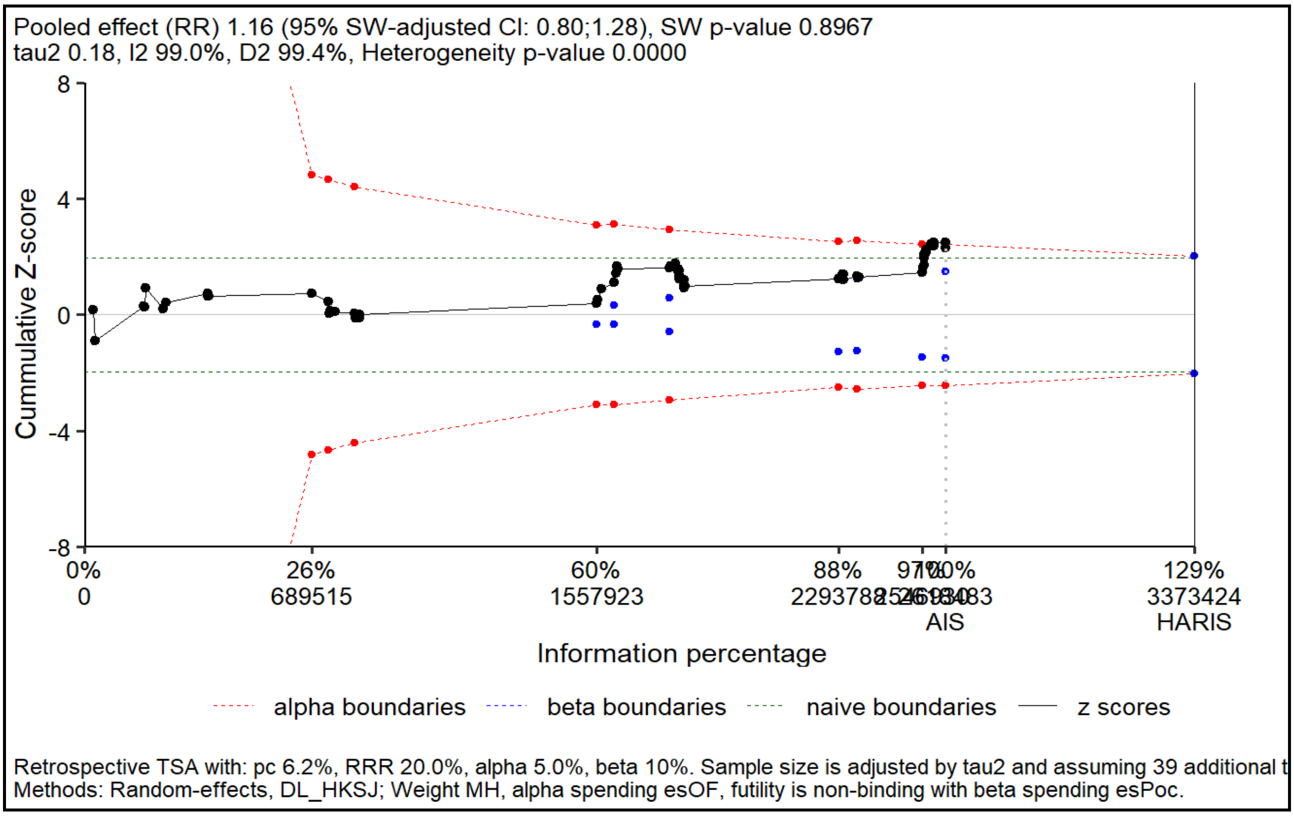

Pooled effect (RR) 1.16 (95% SW-adjusted CI: 0.80;1.28), SW p-value 0.8967
tau2 0.18, I2 99.0%, D2 99.4%, Heterogeneity p-value 0.0000
Cummulative Z-score
8
4
0
-4
-8
0%
0
26%
689515
60%
1557923
88%
2293788
97%
100%
2546083
AIS
129%
3373424
HARIS
Information percentage
alpha boundaries
beta boundaries
naive boundaries
z scores
Retrospective TSA with: pc 6.2%, RRR 20.0%, alpha 5.0%, beta 10%. Sample size is adjusted by tau2 and assuming 39 additional t
Methods: Random-effects, DL_HKSJ; Weight MH, alpha spending esOF, futility is non-binding with beta spending esPoc.

Figure 14S

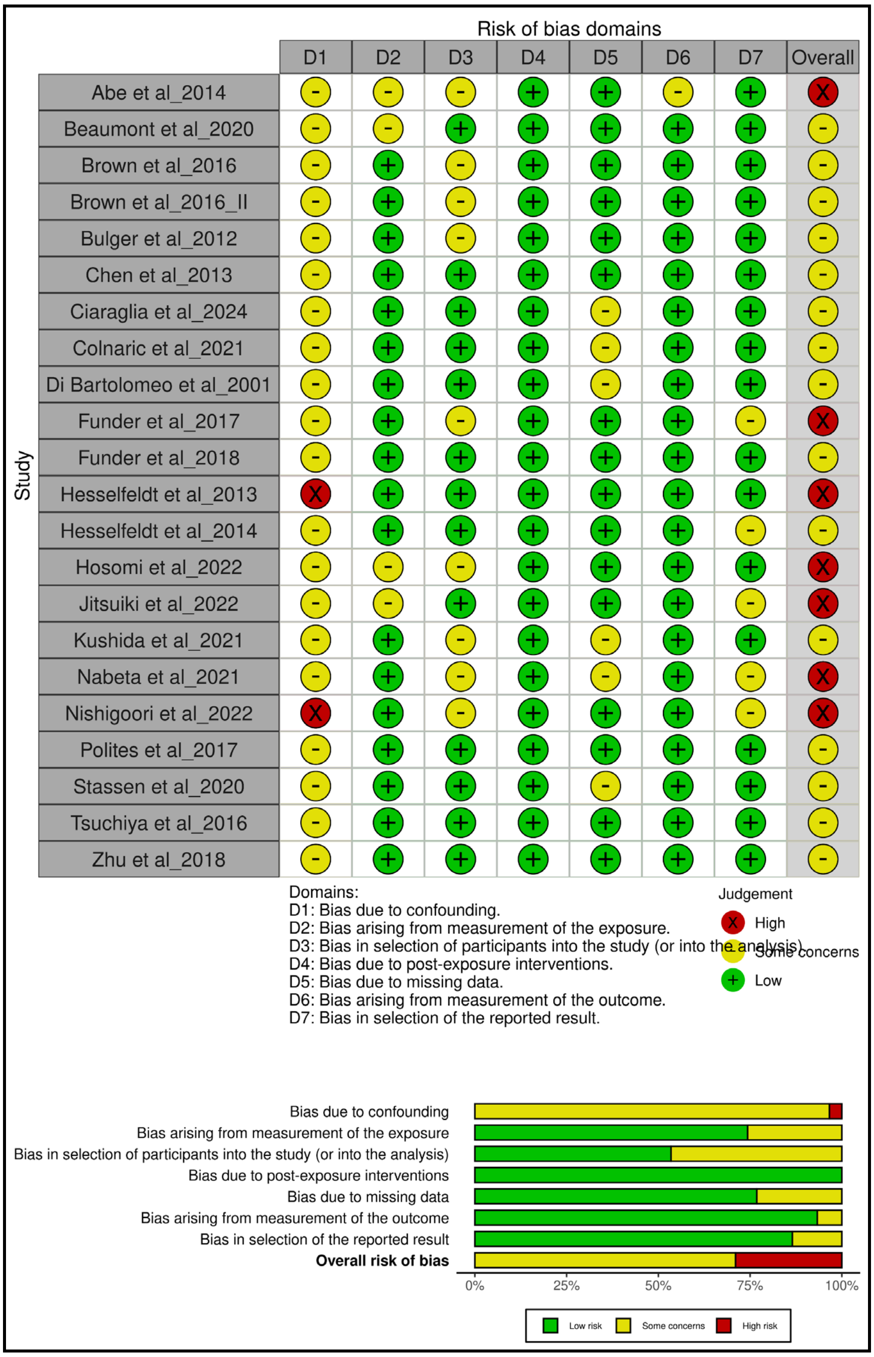
Risk of bias domains
D1
D2
D3
D4
D5
D6
D7
Overall
Study
Abe et al_2014
Beaumont et al_2020
Brown et al_2016
Brown et al_2016_II
Bulger et al_2012
Chen et al_2013
Ciaraglia et al_2024
Colnaric et al_2021
Di Bartolomeo et al_2001
Funder et al_2017
Funder et al_2018
Hesselfeldt et al_2013
Hesselfeldt et al_2014
Hosomi et al_2022
Jitsuiki et al_2022
Kushida et al_2021
Nabeta et al_2021
Nishigoori et al_2022
Polites et al_2017
Stassen et al_2020
Tsuchiya et al_2016
Zhu et al_2018
Domains:
D1: Bias due to confounding.
D2: Bias arising from measurement of the exposure.
D3: Bias in selection of participants into the study (or into the analysis).
D4: Bias due to post-exposure interventions.
D5: Bias due to missing data.
D6: Bias arising from measurement of the outcome.
D7: Bias in selection of the reported result.
Judgement
High
Some concerns
Low
Bias due to confounding
Bias arising from measurement of the exposure
Bias in selection of participants into the study (or into the analysis)
Bias due to post-exposure interventions
Bias due to missing data
Bias arising from measurement of the outcome
Bias in selection of the reported result
Overall risk of bias
0%
25%
50%
75%
100%
Low risk
Some concerns
High risk

Figure 15S

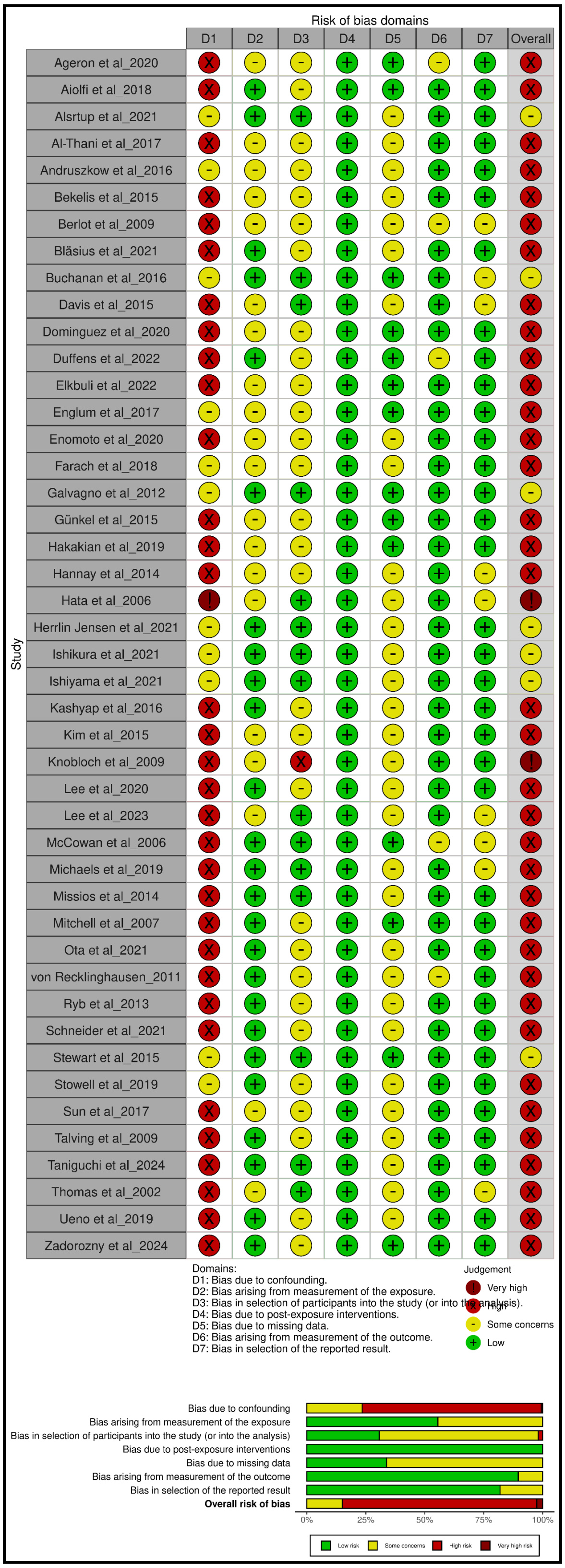

Risk of bias domains
D1
D2
D3
D4
D5
D6
D7
Overall
Study
Ageron et al_2020
Aiolfi et al_2018
Alsrtup et al_2021
Al-Thani et al_2017
Andruszkow et al_2016
Bekelis et al_2015
Berlot et al_2009
Bläsius et al_2021
Buchanan et al_2016
Davis et al_2015
Dominguez et al_2020
Duffens et al_2022
Elkbuli et al_2022
Englum et al_2017
Enomoto et al_2020
Farach et al_2018
Galvagno et al_2012
Günkel et al_2015
Hakakian et al_2019
Hannay et al_2014
Hata et al_2006
Herrlin Jensen et al_2021
Ishikura et al_2021
Ishiyama et al_2021
Kashyap et al_2016
Kim et al_2015
Knobloch et al_2009
Lee et al_2020
Lee et al_2023
McCowan et al_2006
Michaels et al_2019
Missios et al_2014
Mitchell et al_2007
Ota et al_2021
von Recklinghausen_2011
Ryb et al_2013
Schneider et al_2021
Stewart et al_2015
Stowell et al_2019
Sun et al_2017
Talving et al_2009
Taniguchi et al_2024
Thomas et al_2002
Ueno et al_2019
Zadorozny et al_2024
Domains:
D1: Bias due to confounding.
D2: Bias arising from measurement of the exposure.
D3: Bias in selection of participants into the study (or into the analysis).
D4: Bias due to post-exposure interventions.
D5: Bias due to missing data.
D6: Bias arising from measurement of the outcome.
D7: Bias in selection of the reported result.
Judgement
Very high
High
Some concerns
Low
Bias due to confounding
Bias arising from measurement of the exposure
Bias in selection of participants into the study (or into the analysis)
Bias due to post-exposure interventions
Bias due to missing data
Bias arising from measurement of the outcome
Bias in selection of the reported result
Overall risk of bias
0%
25%
50%
75%
100%
Low risk
Some concerns
High risk
Very high risk

Figure 16S

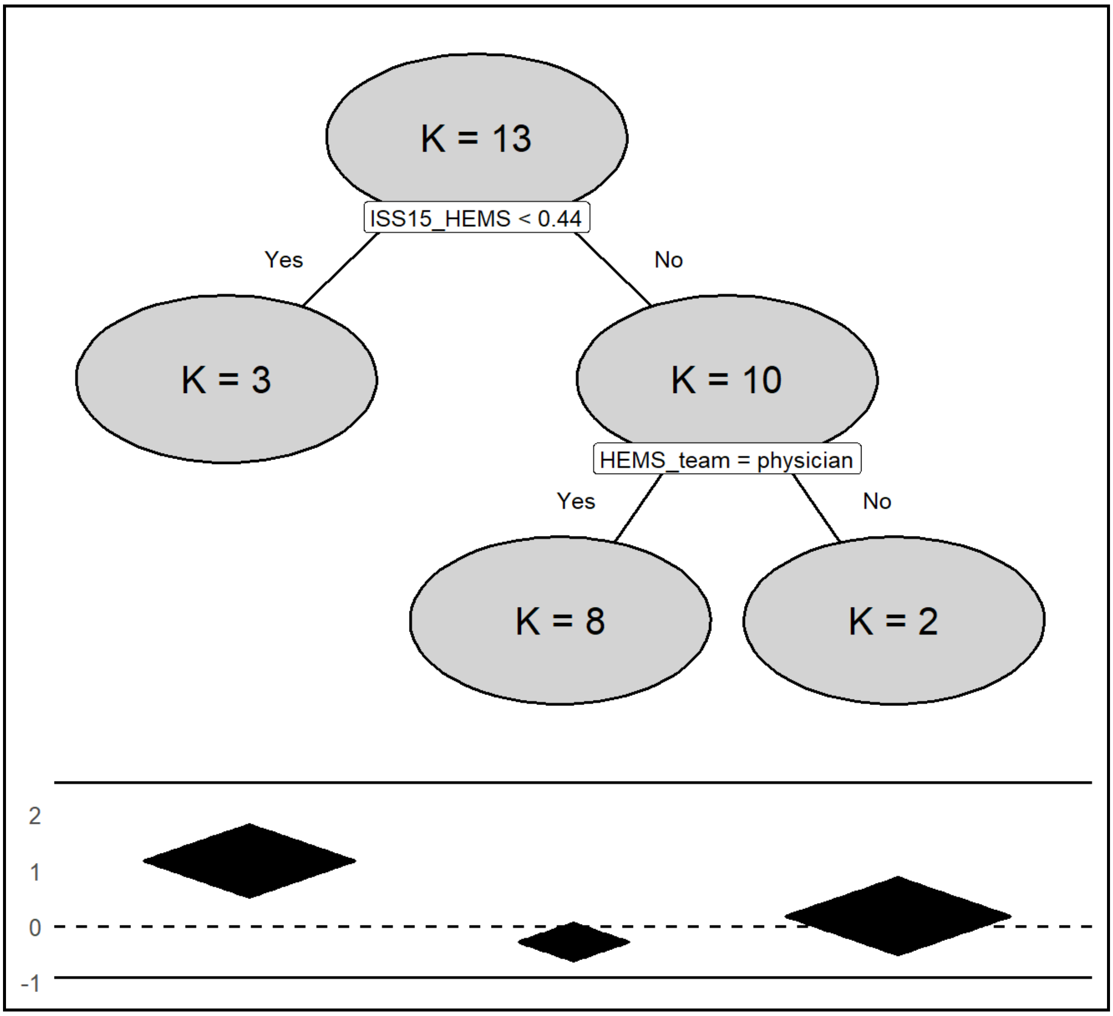

K = 13
ISS15_HEMS < 0.44
Yes
No
K = 3
K = 10
HEMS_team = physician
Yes
No
K = 8
K = 2
2
1
0
-1

Figure 17S

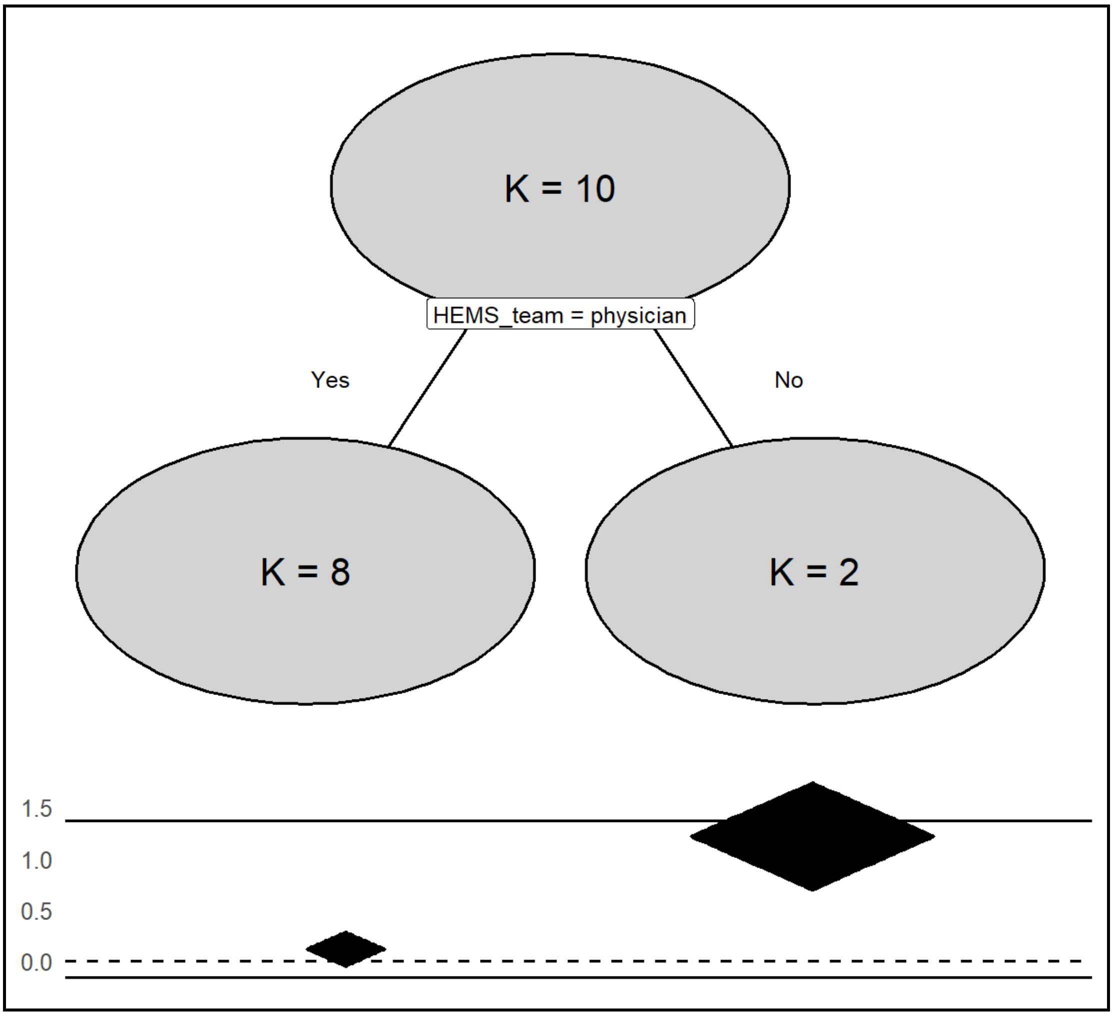

Supplement: Supplementary file 1 — Supplementary Material 1 [file 13049_2025_1478_MOESM1_ESM.pdf]
